# Supplementary material for: A strategy to find novel candidate anti-Alzheimer’s disease drugs by constructing interaction networks between drug targets and natural compounds in medical plants
Source: PeerJ. 2018 May 11;6:e4756. doi: 10.7717/peerj.4756 (PMC5951129; doi:10.7717/peerj.4756)
Supplement: Figure 1–34 [file peerj-06-4756-s035.pdf]

SUPPLEMENTARY MATERIAL

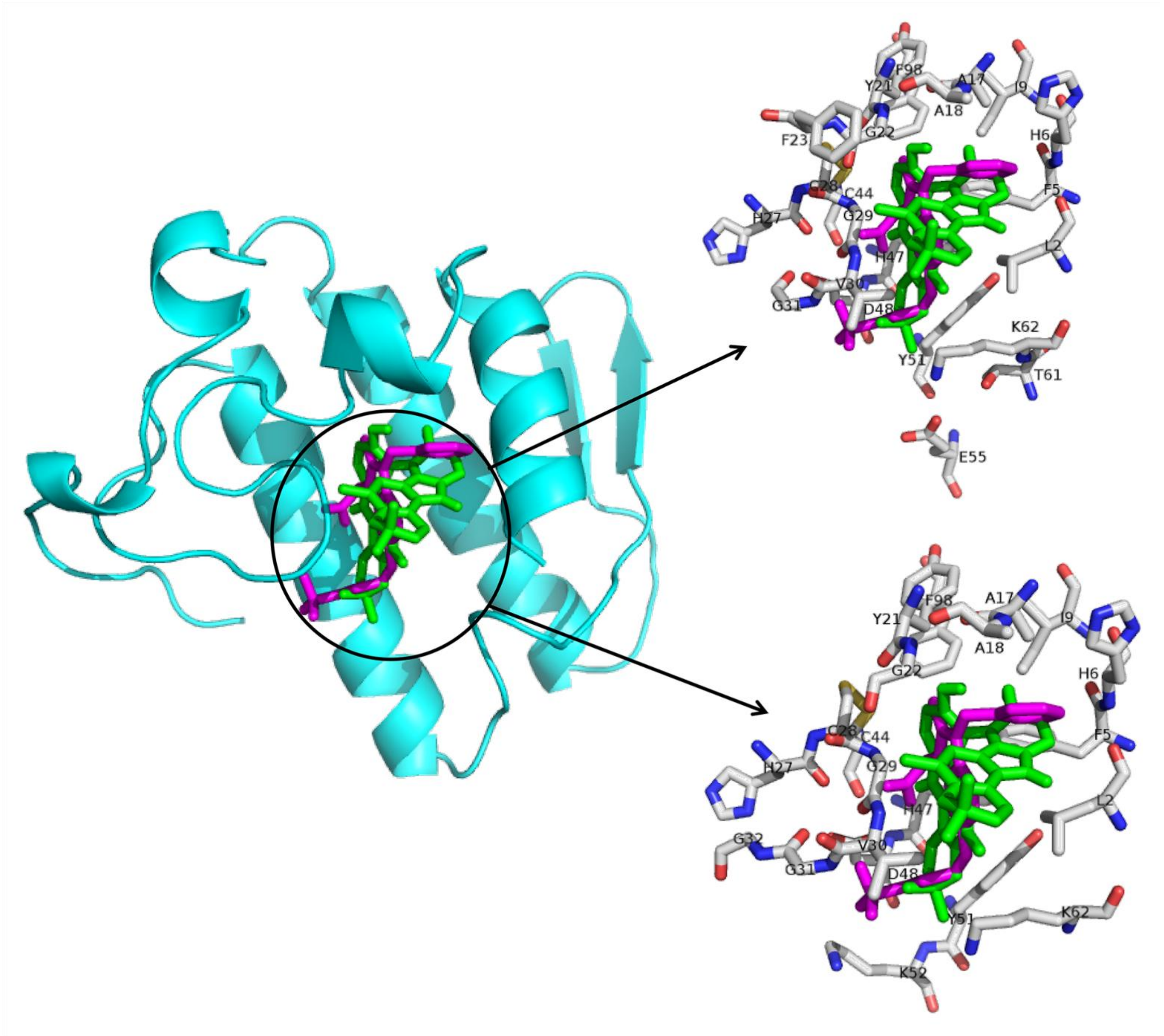

Supplementary Fig 1. The structures and binding model of ligands including best ligand and original ligand to the target protein PLA2. The best ligand is green and the original ligand is magenta. The top panel shows the amino acid residues lying within 5 Å from the best ligand, and the bottom panel shows the amino acid residues lying within 5 Å from the original ligand.

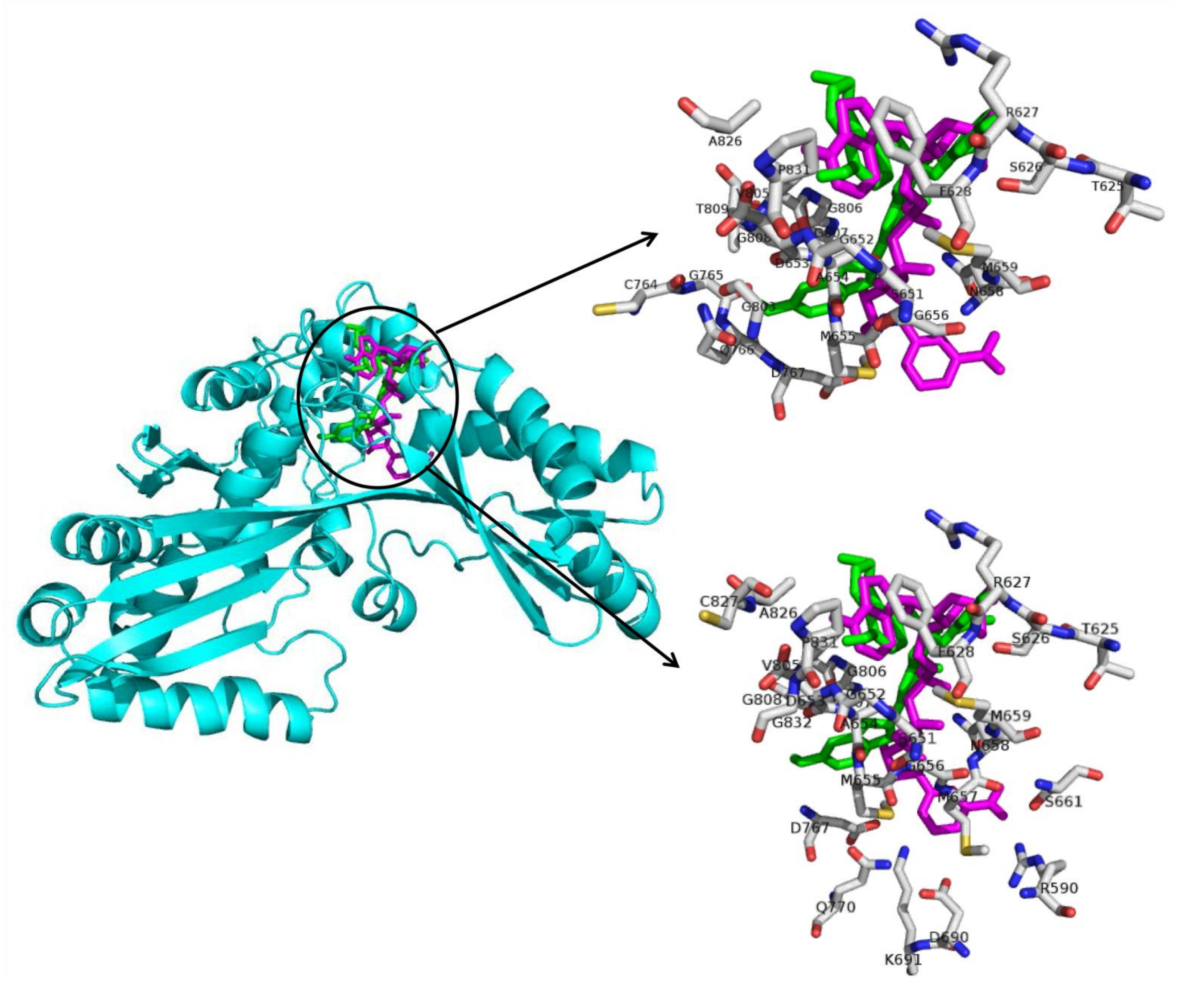

Supplementary Fig 2. The structures and binding model of ligands including best ligand and original ligand to the target protein HMG-COA. The best ligand is green and the original ligand is magenta. The top panel shows the amino acid residues lying within 5 Å from the best ligand, and the bottom panel shows the amino acid residues lying within 5 Å from the original ligand.

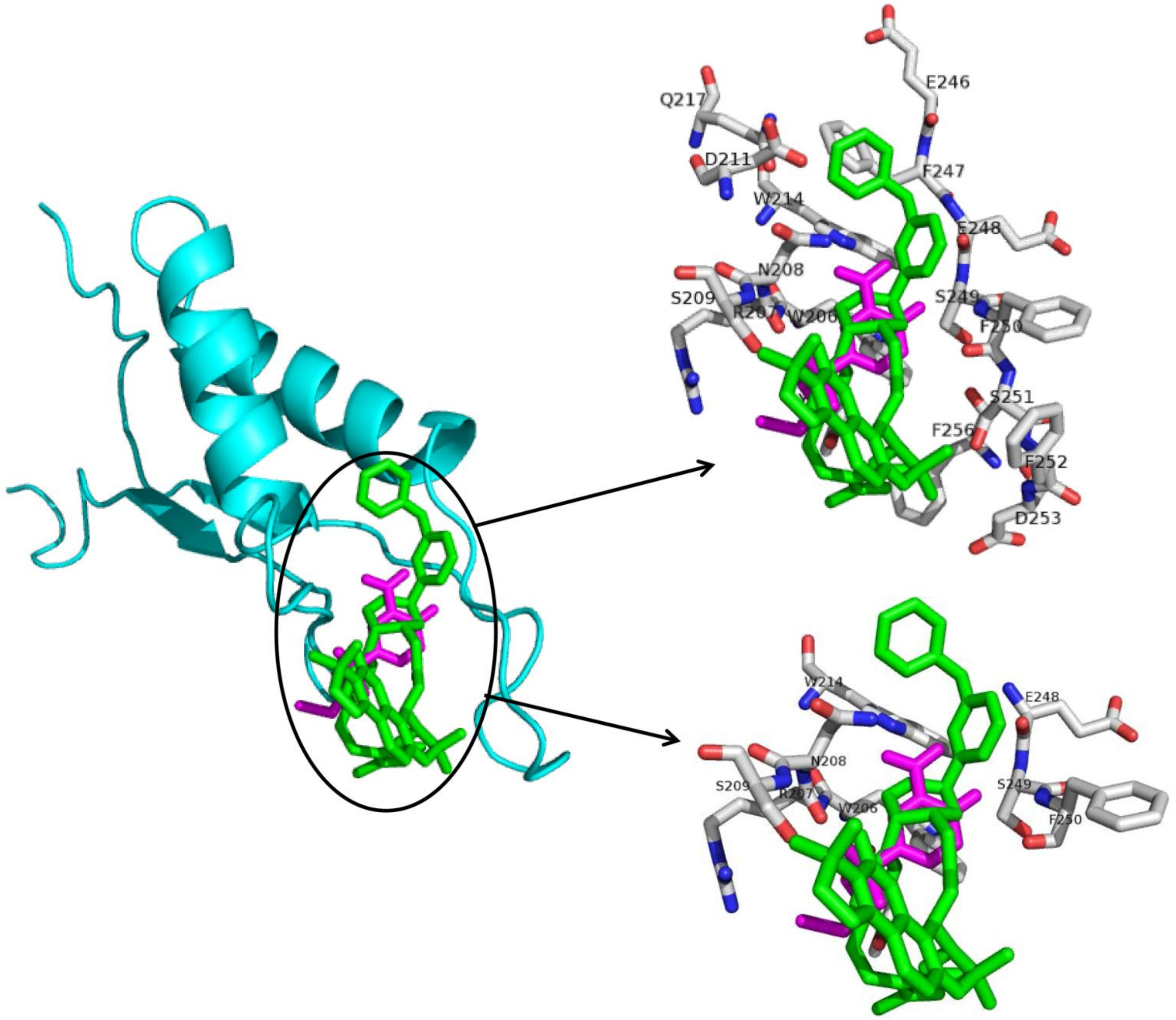

Supplementary Fig 3. The structures and binding model of ligands including best ligand and original ligand to the target protein Caspase-3. The best ligand is green and the original ligand is magenta. The top panel shows the amino acid residues lying within 5 Å from the best ligand, and the bottom panel shows the amino acid residues lying within 5 Å from the original ligand.

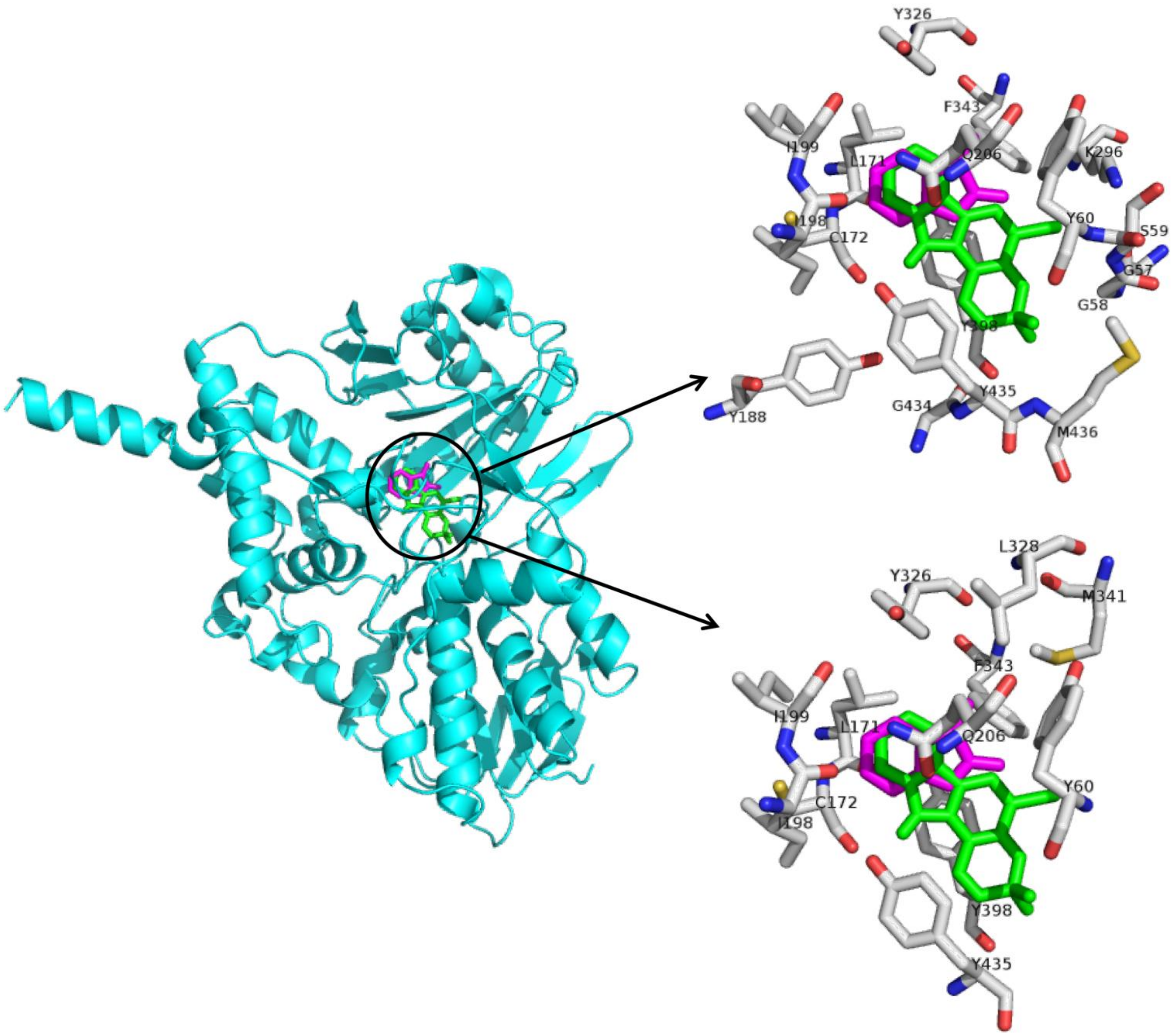

Supplementary Fig 4. The structures and binding model of ligands including best ligand and original ligand to the target protein MAOB. The best ligand is green and the original ligand is magenta. The top panel shows the amino acid residues lying within 5 Å from the best ligand, and the bottom panel shows the amino acid residues lying within 5 Å from the original ligand.

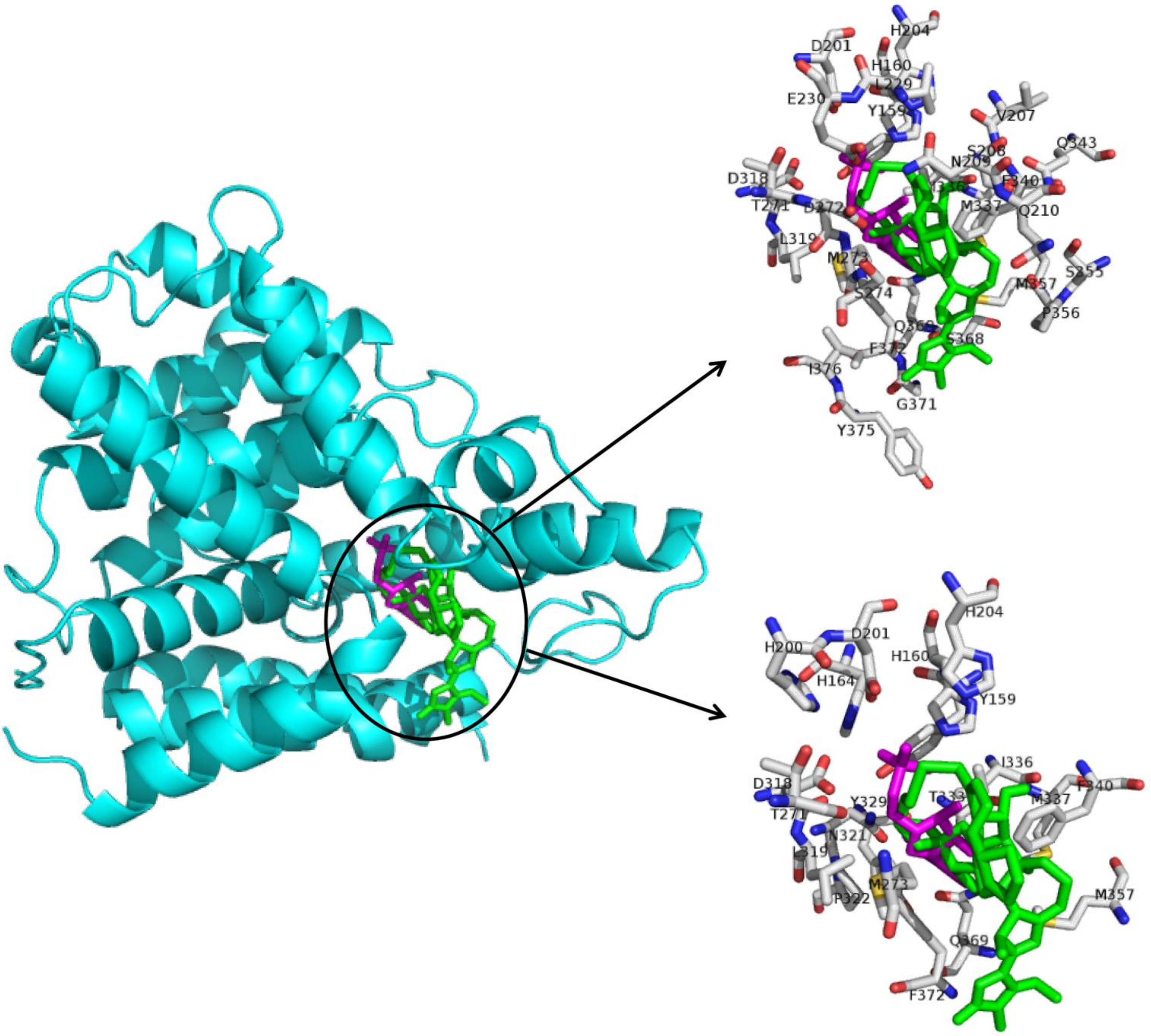

Supplementary Fig 5. The structures and binding model of ligands including best ligand and original ligand to the target protein PDE4. The best ligand is green and the original ligand is magenta. The top panel shows the amino acid residues lying within 5 Å from the best ligand, and the bottom panel shows the amino acid residues lying within 5 Å from the original ligand.

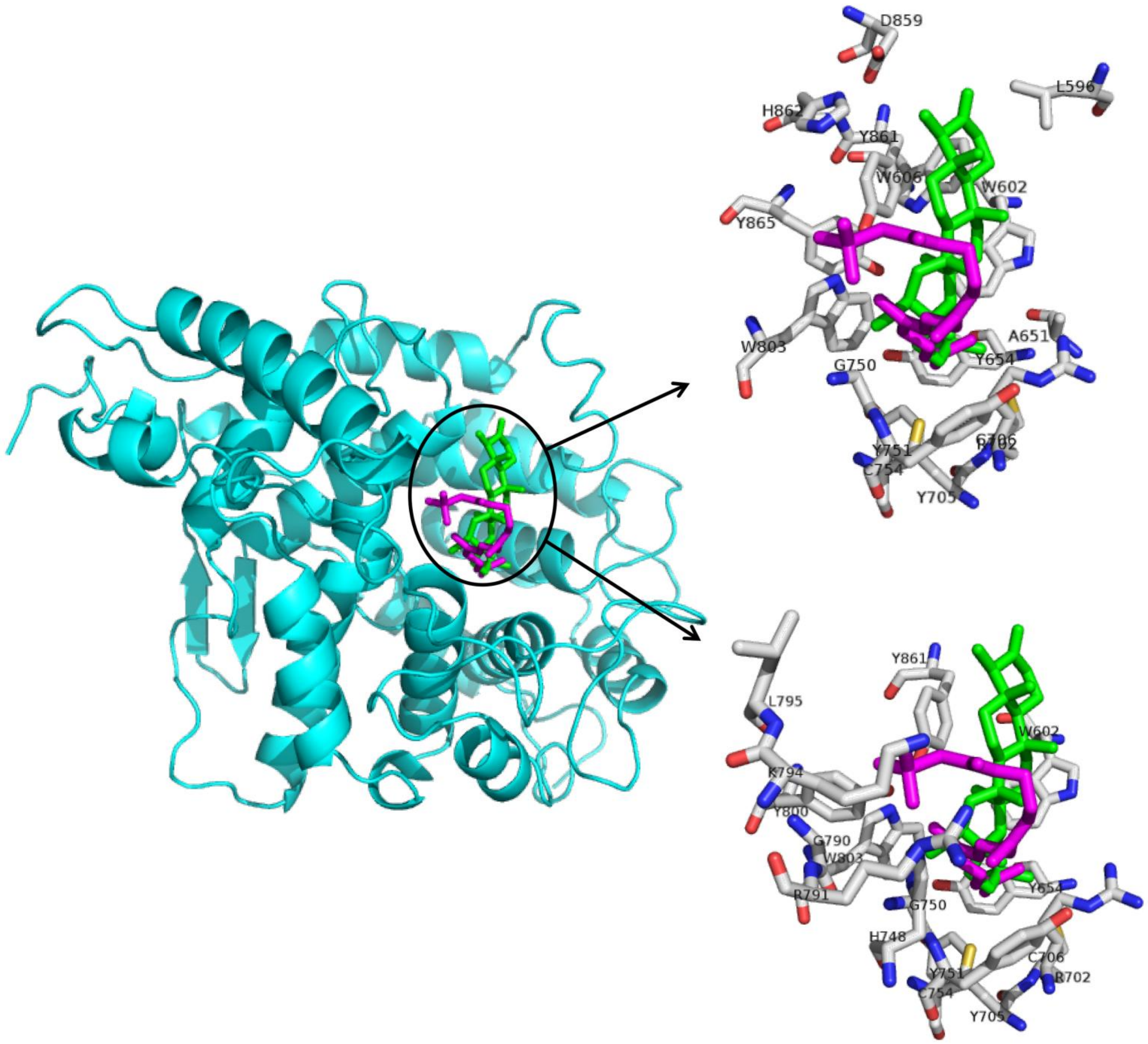

Supplementary Fig 6. The structures and binding model of ligands including best ligand and original ligand to the target protein Ftase. The best ligand is green and the original ligand is magenta. The top panel shows the amino acid residues lying within 5 Å from the best ligand, and the bottom panel shows the amino acid residues lying within 5 Å from the original ligand.

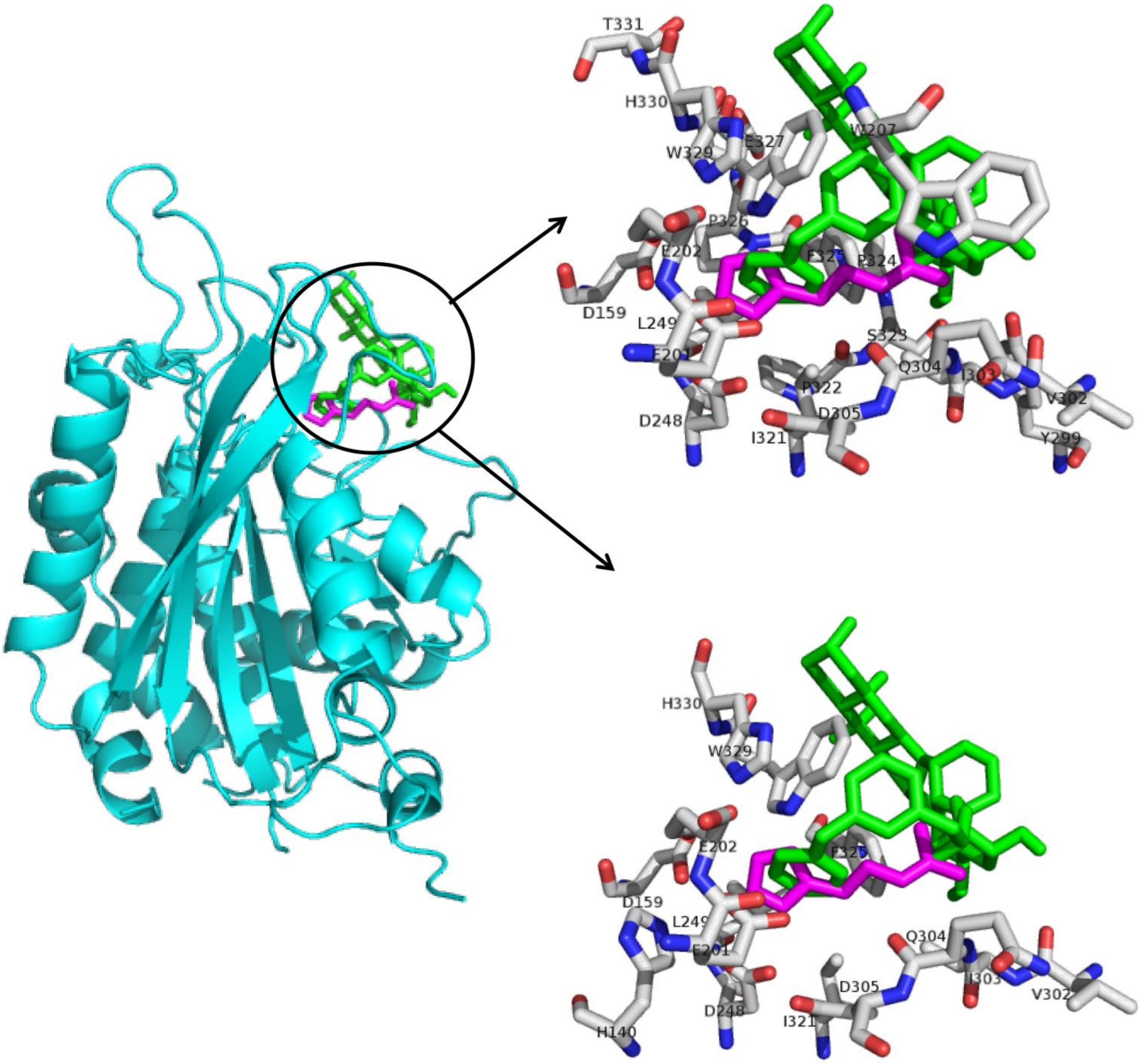

Supplementary Fig 7. The structures and binding model of ligands including best ligand and original ligand to the target protein QC. The best ligand is green and the original ligand is magenta. The top panel shows the amino acid residues lying within 5 Å from the best ligand, and the bottom panel shows the amino acid residues lying within 5 Å from the original ligand.

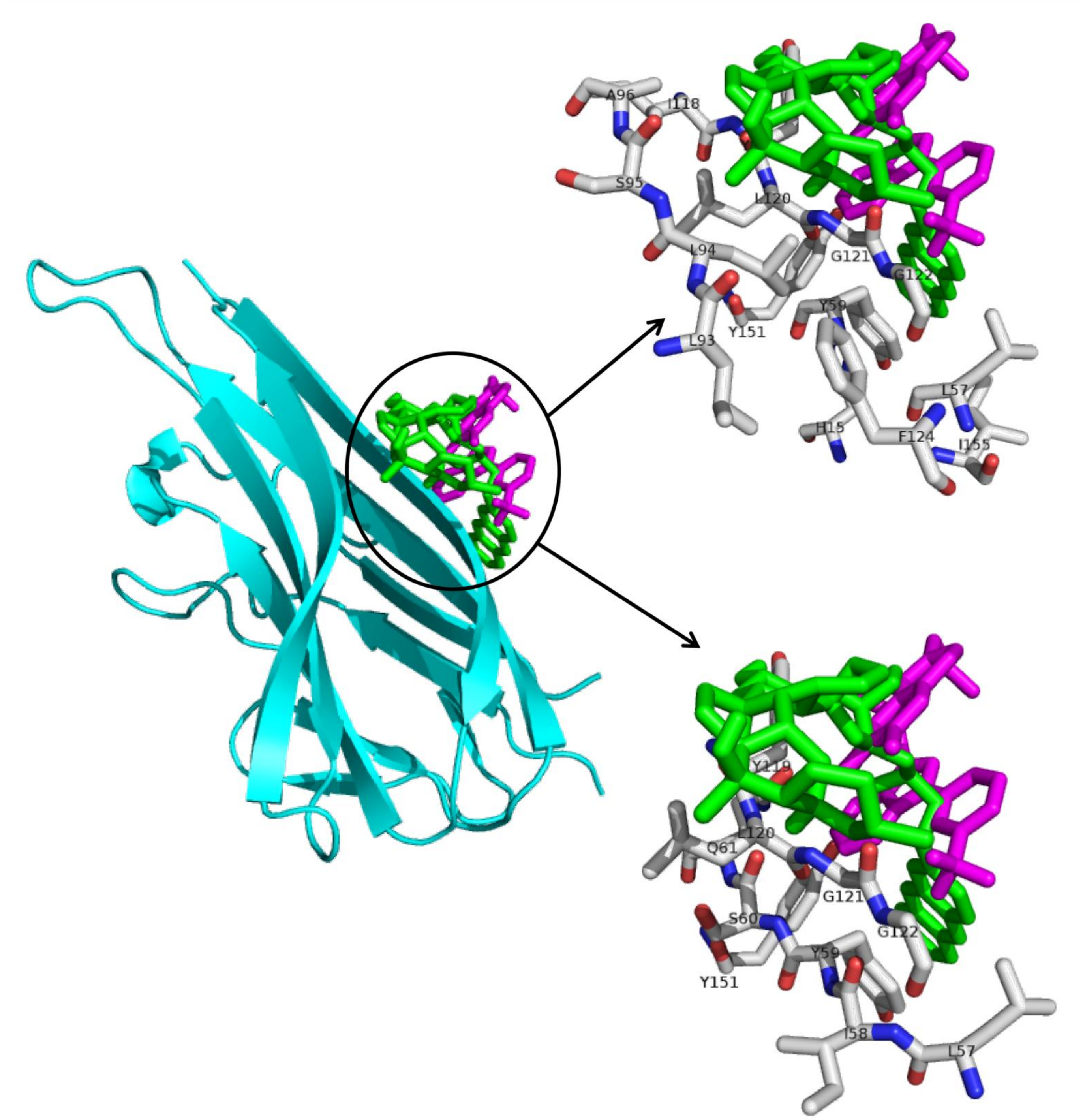

Supplementary Fig 8. The structures and binding model of ligands including best ligand and original ligand to the target protein TNF. The best ligand is green and the original ligand is magenta. The top panel shows the amino acid residues lying within 5 Å from the best ligand, and the bottom panel shows the amino acid residues lying within 5 Å from the original ligand.

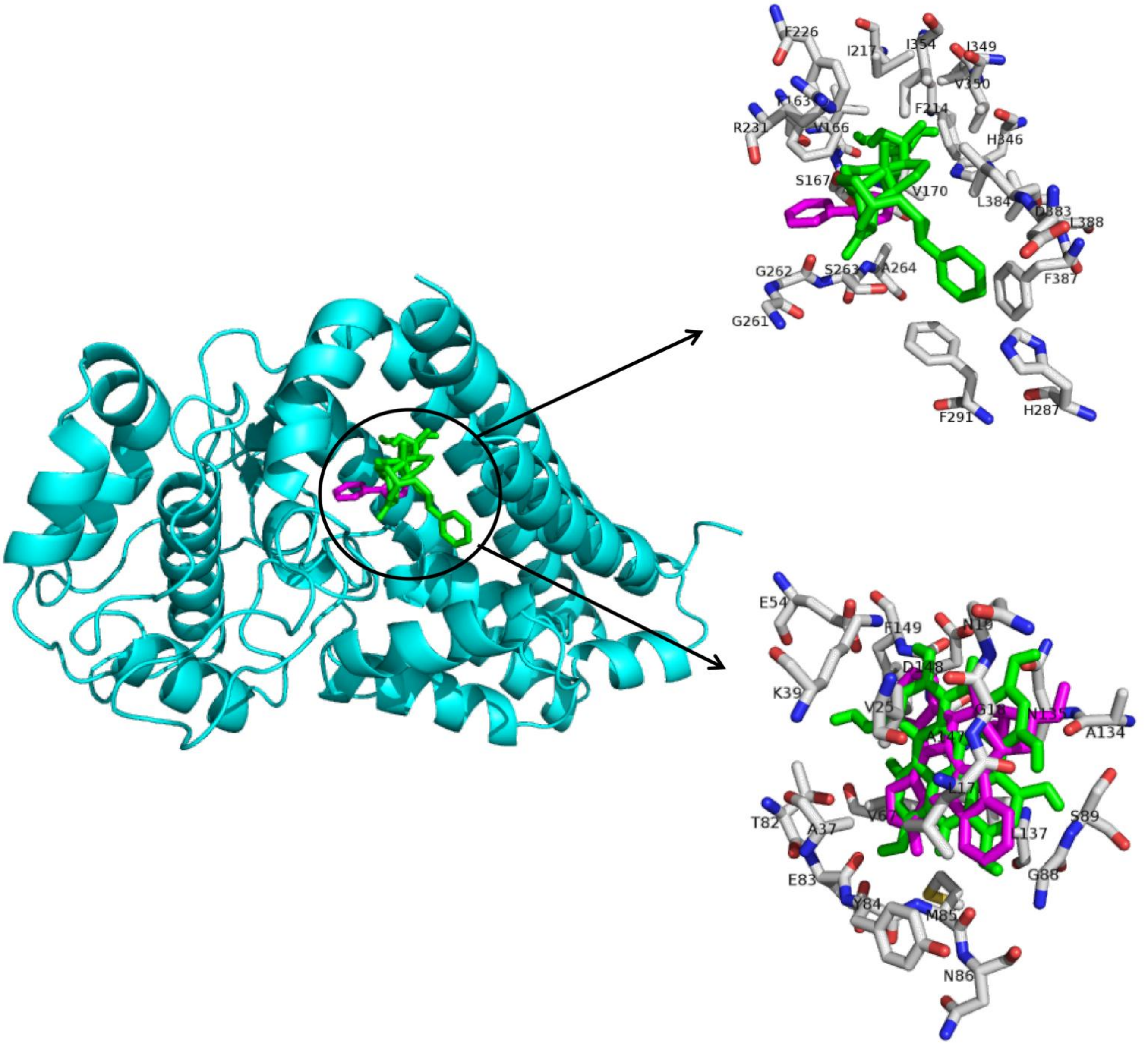

Supplementary Fig 9. The structures and binding model of ligands including best ligand and original ligand to the target protein IDO. The best ligand is green and the original ligand is magenta. The top panel shows the amino acid residues lying within 5 Å from the best ligand, and the bottom panel shows the amino acid residues lying within 5 Å from the original ligand.

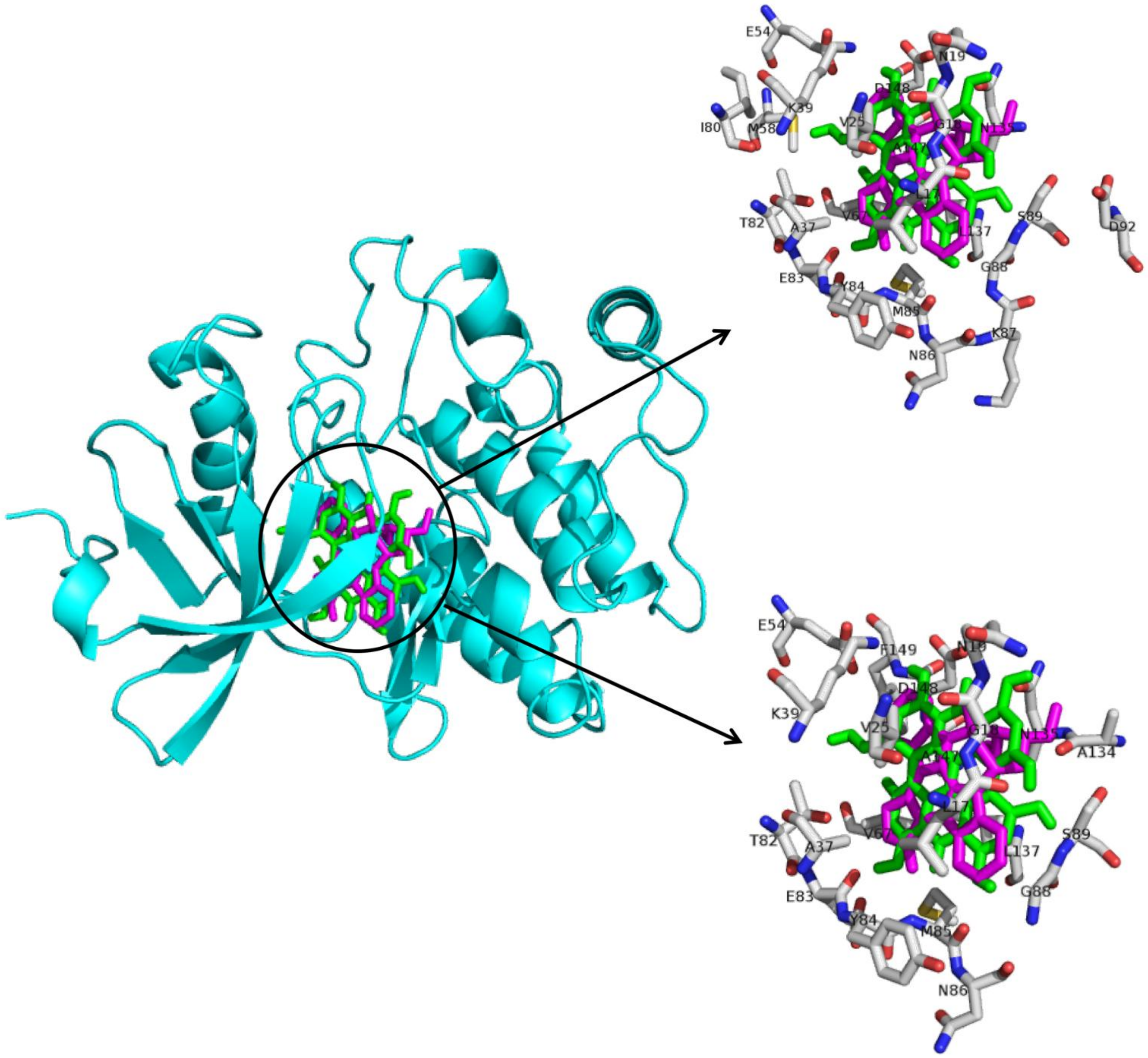

Supplementary Fig 10. The structures and binding model of ligands including best ligand and original ligand to the target protein Fyn. The best ligand is green and the original ligand is magenta. The top panel shows the amino acid residues lying within 5 Å from the best ligand, and the bottom panel shows the amino acid residues lying within 5 Å from the original ligand.

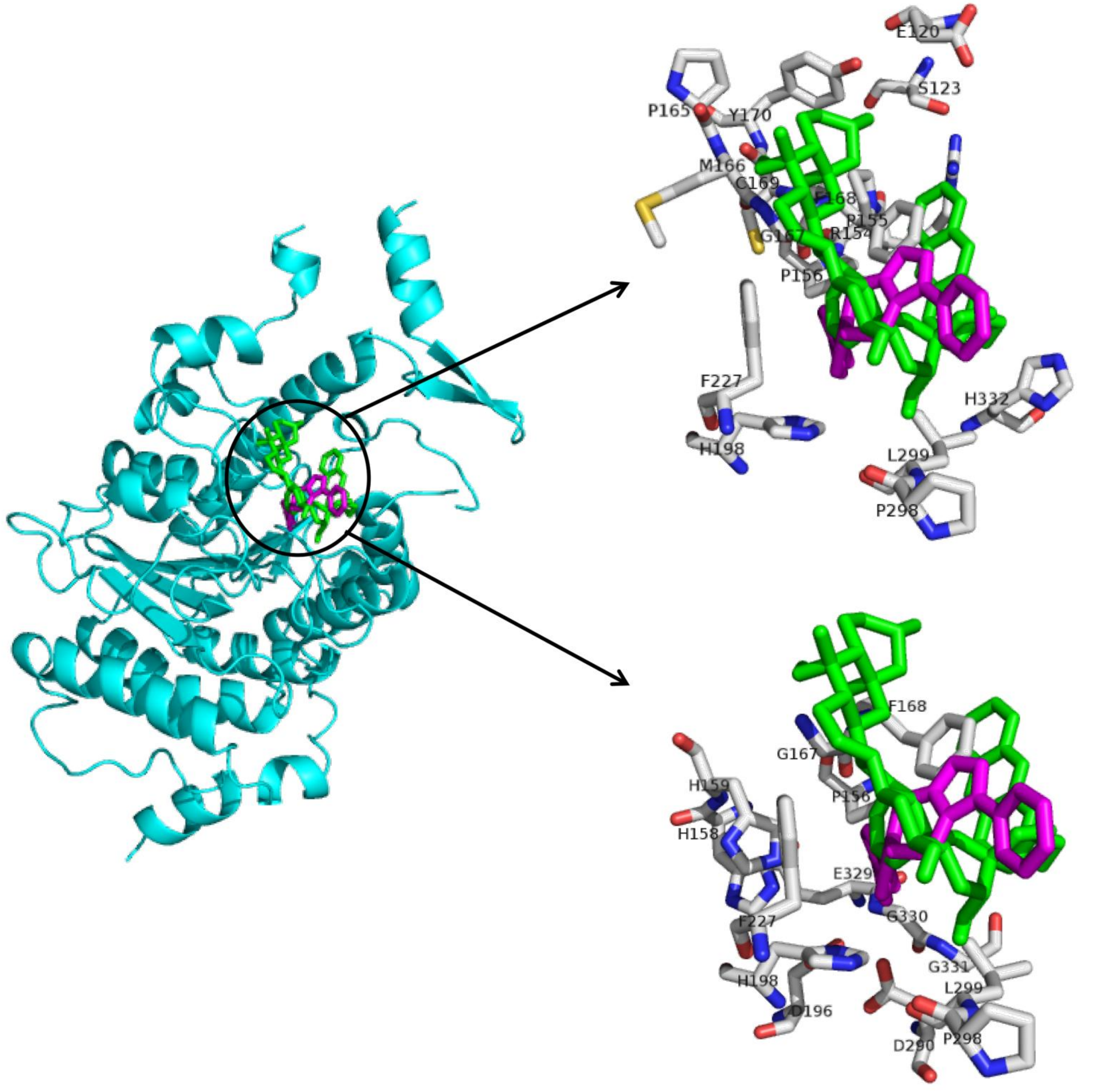

Supplementary Fig 11. The structures and binding model of ligands including best ligand and original ligand to the target protein HDAC. The best ligand is green and the original ligand is magenta. The top panel shows the amino acid residues lying within 5 Å from the best ligand, and the bottom panel shows the amino acid residues lying within 5 Å from the original ligand.

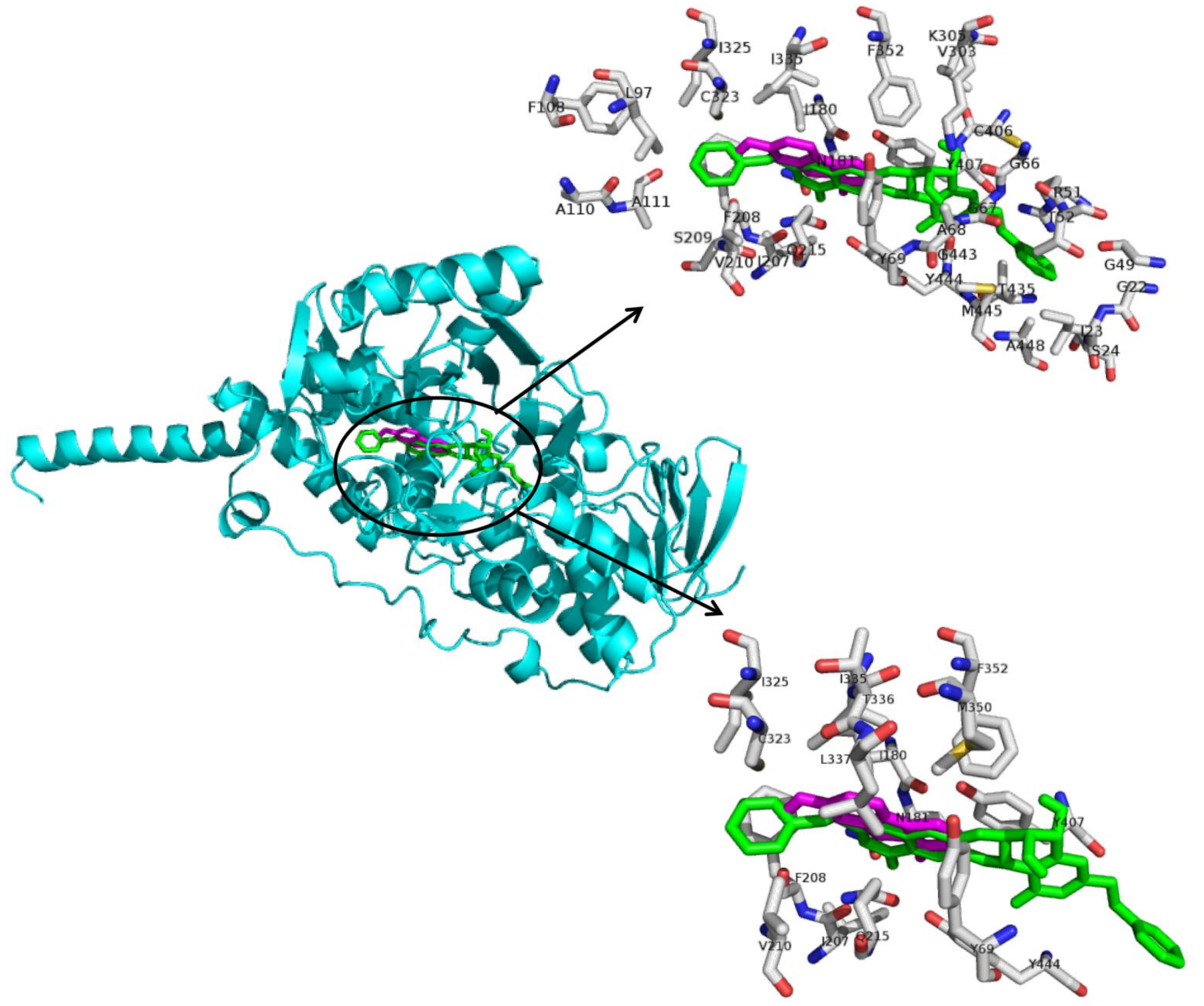

Supplementary Fig 12. The structures and binding model of ligands including best ligand and original ligand to the target protein MAOA. The best ligand is green and the original ligand is magenta. The top panel shows the amino acid residues lying within 5 Å from the best ligand, and the bottom panel shows the amino acid residues lying within 5 Å from the original ligand.

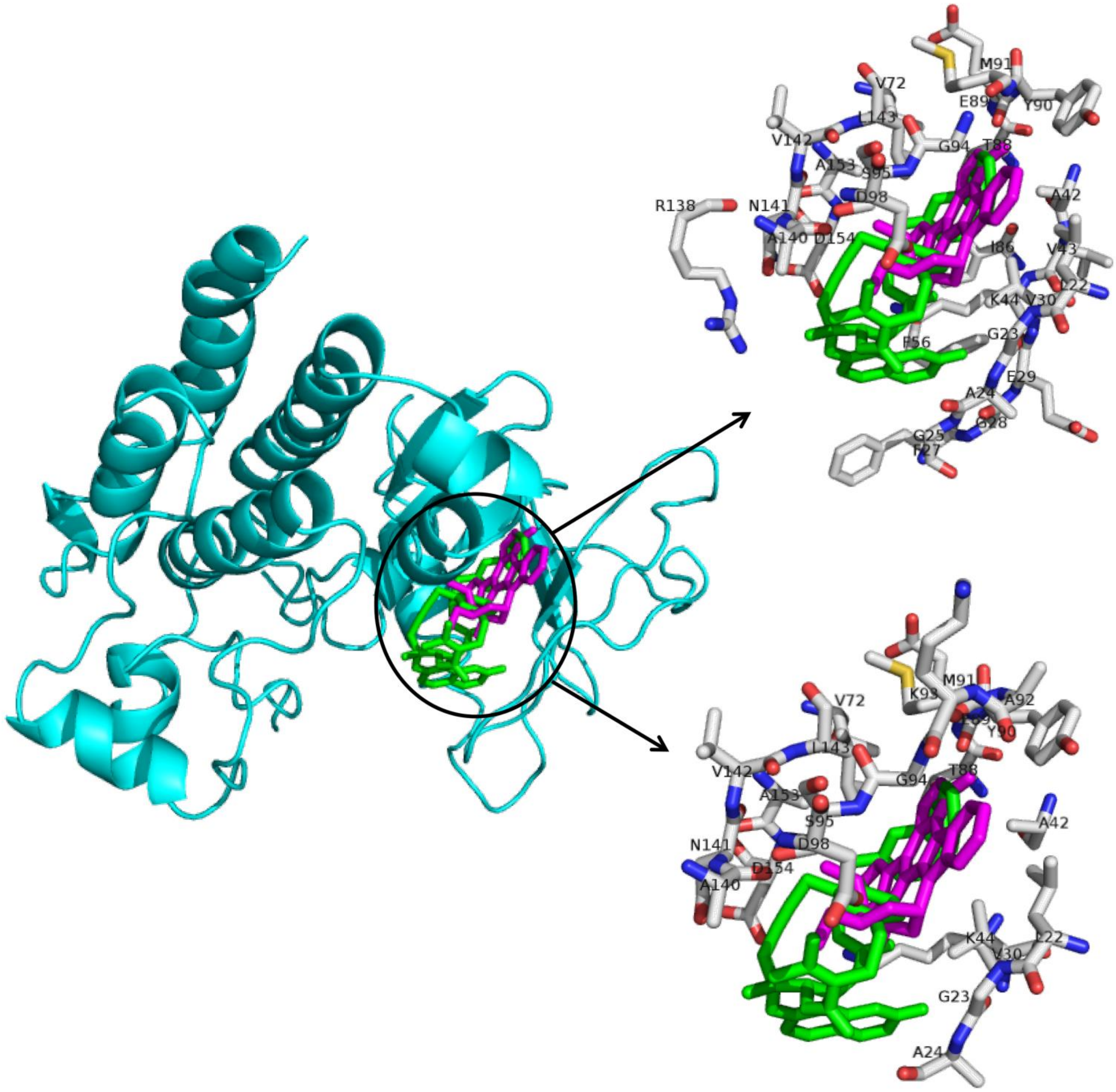

Supplementary Fig 13. The structures and binding model of ligands including best ligand and original ligand to the target protein lyn. The best ligand is green and the original ligand is magenta. The top panel shows the amino acid residues lying within 5 Å from the best ligand, and the bottom panel shows the amino acid residues lying within 5 Å from the original ligand.

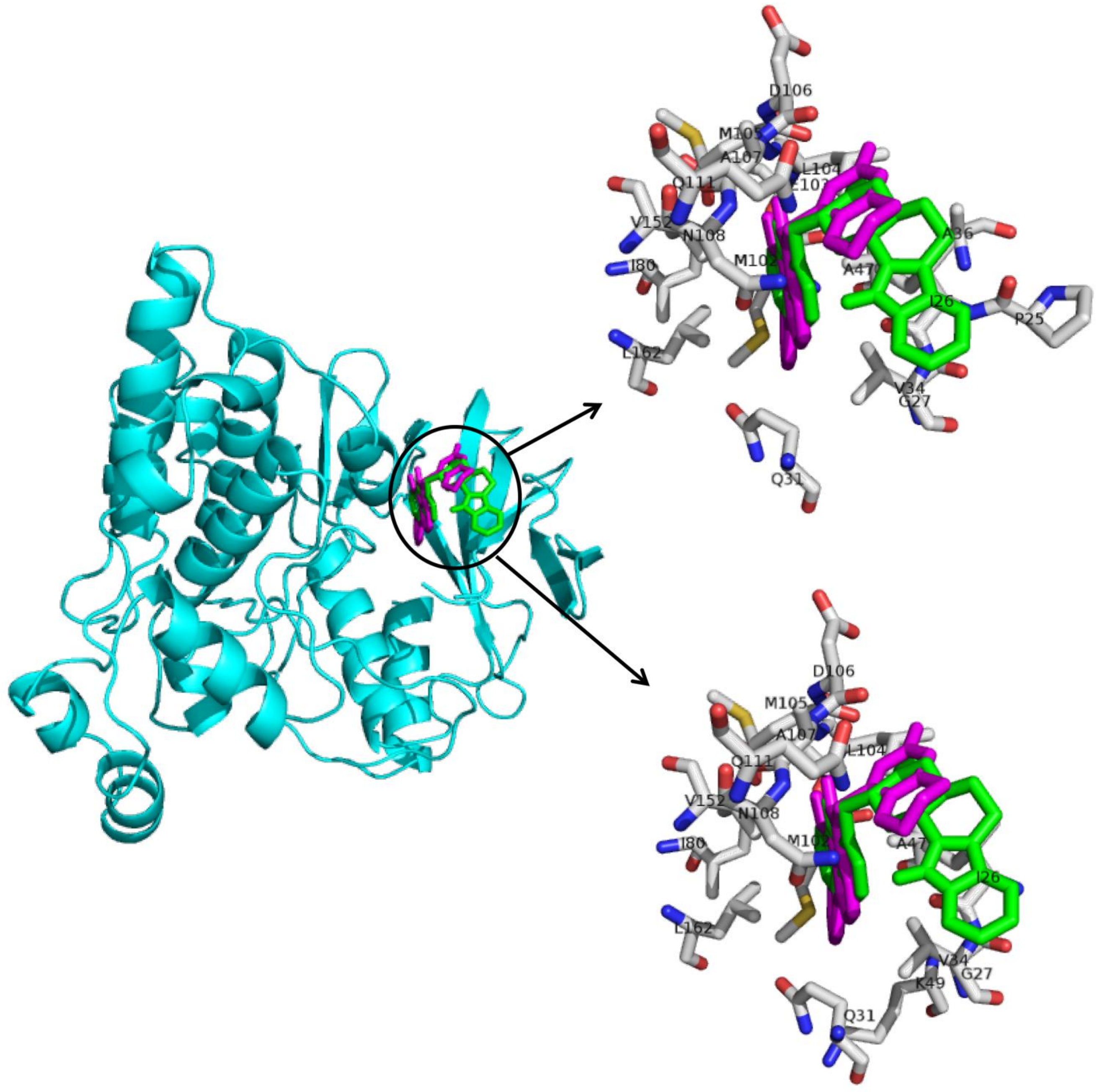

Supplementary Fig 14. The structures and binding model of ligands including best ligand and original ligand to the target protein JNK. The best ligand is green and the original ligand is magenta. The top panel shows the amino acid residues lying within 5 Å from the best ligand, and the bottom panel shows the amino acid residues lying within 5 Å from the original ligand.

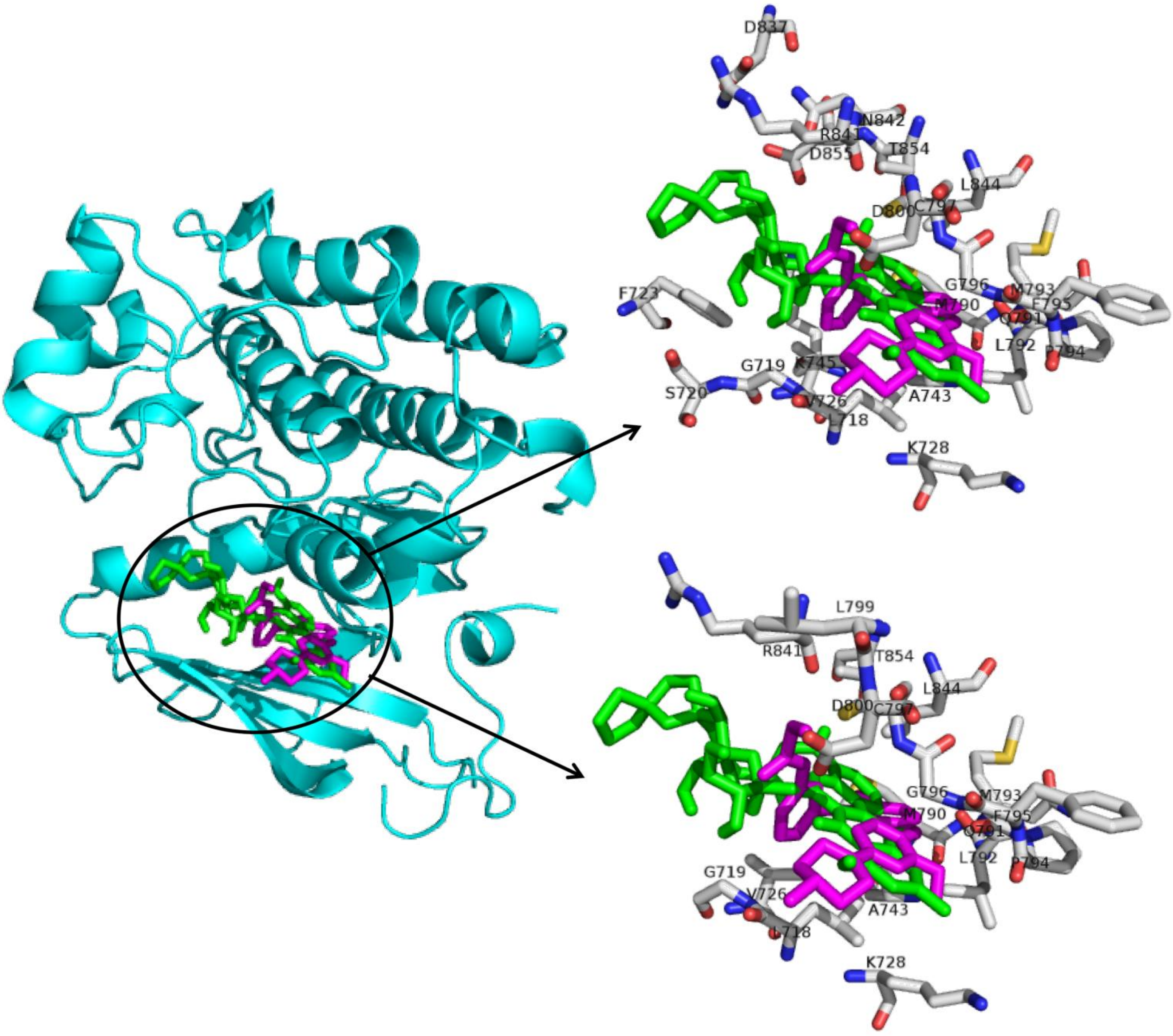

Supplementary Fig 15. The structures and binding model of ligands including best ligand and original ligand to the target protein EGFR. The best ligand is green and the original ligand is magenta. The top panel shows the amino acid residues lying within 5 Å from the best ligand, and the bottom panel shows the amino acid residues lying within 5 Å from the original ligand.

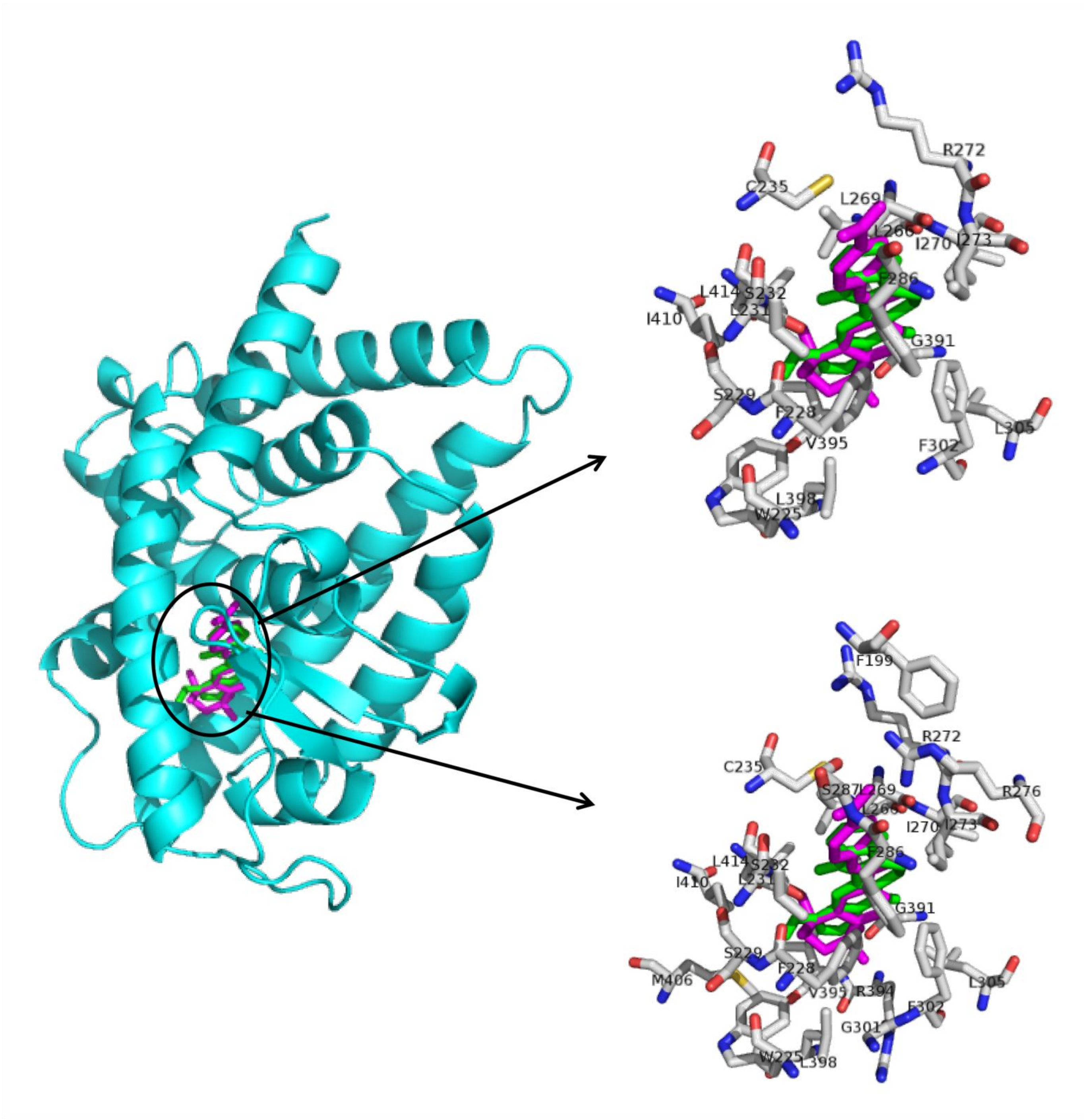

Supplementary Fig 16. The structures and binding model of ligands including best ligand and original ligand to the target protein RAR. The best ligand is green and the original ligand is magenta. The top panel shows the amino acid residues lying within 5 Å from the best ligand, and the bottom panel shows the amino acid residues lying within 5 Å from the original ligand.

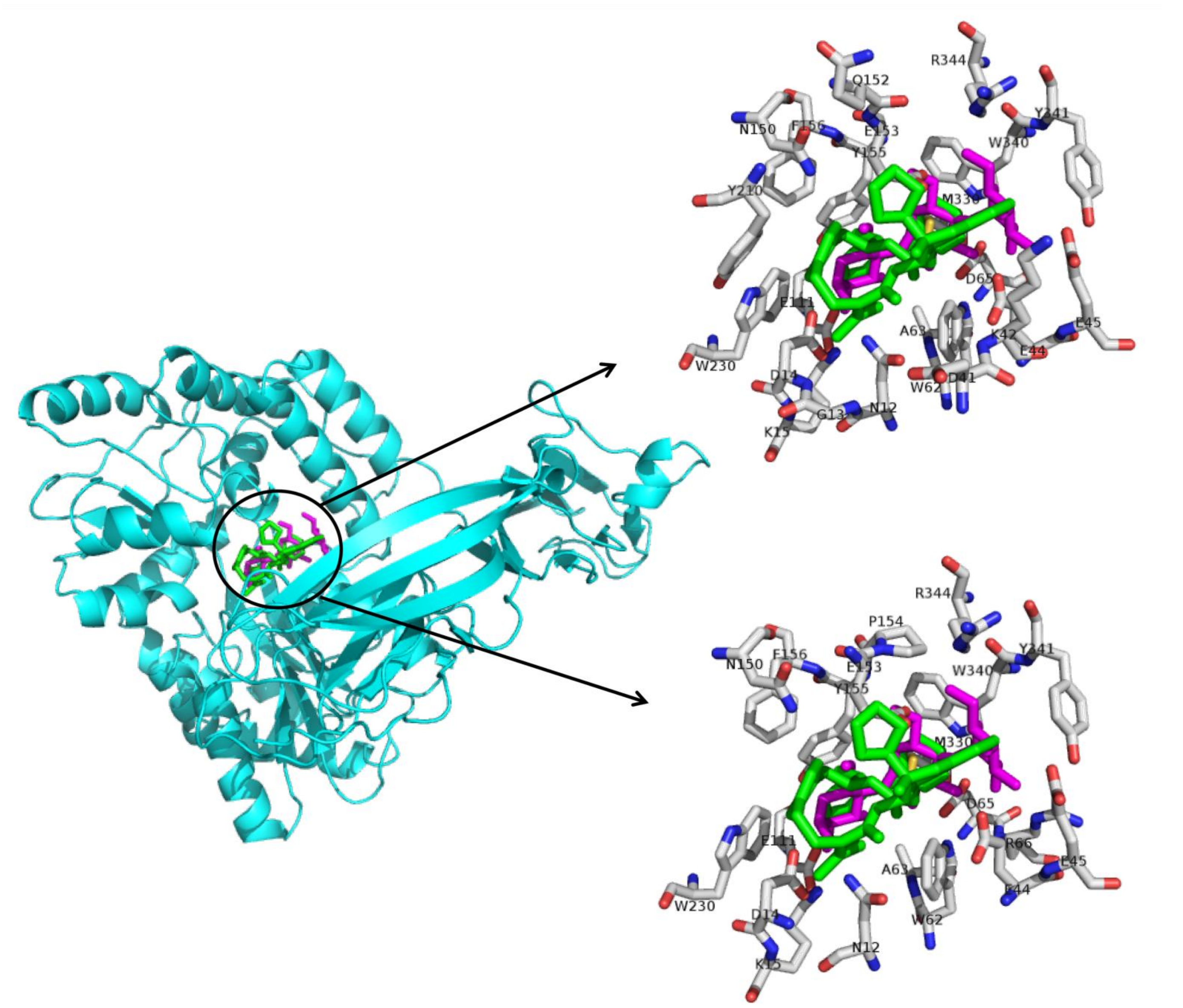

Supplementary Fig 17. The structures and binding model of ligands including best ligand and original ligand to the target protein RAGE. The best ligand is green and the original ligand is magenta. The top panel shows the amino acid residues lying within 5 Å from the best ligand, and the bottom panel shows the amino acid residues lying within 5 Å from the original ligand.

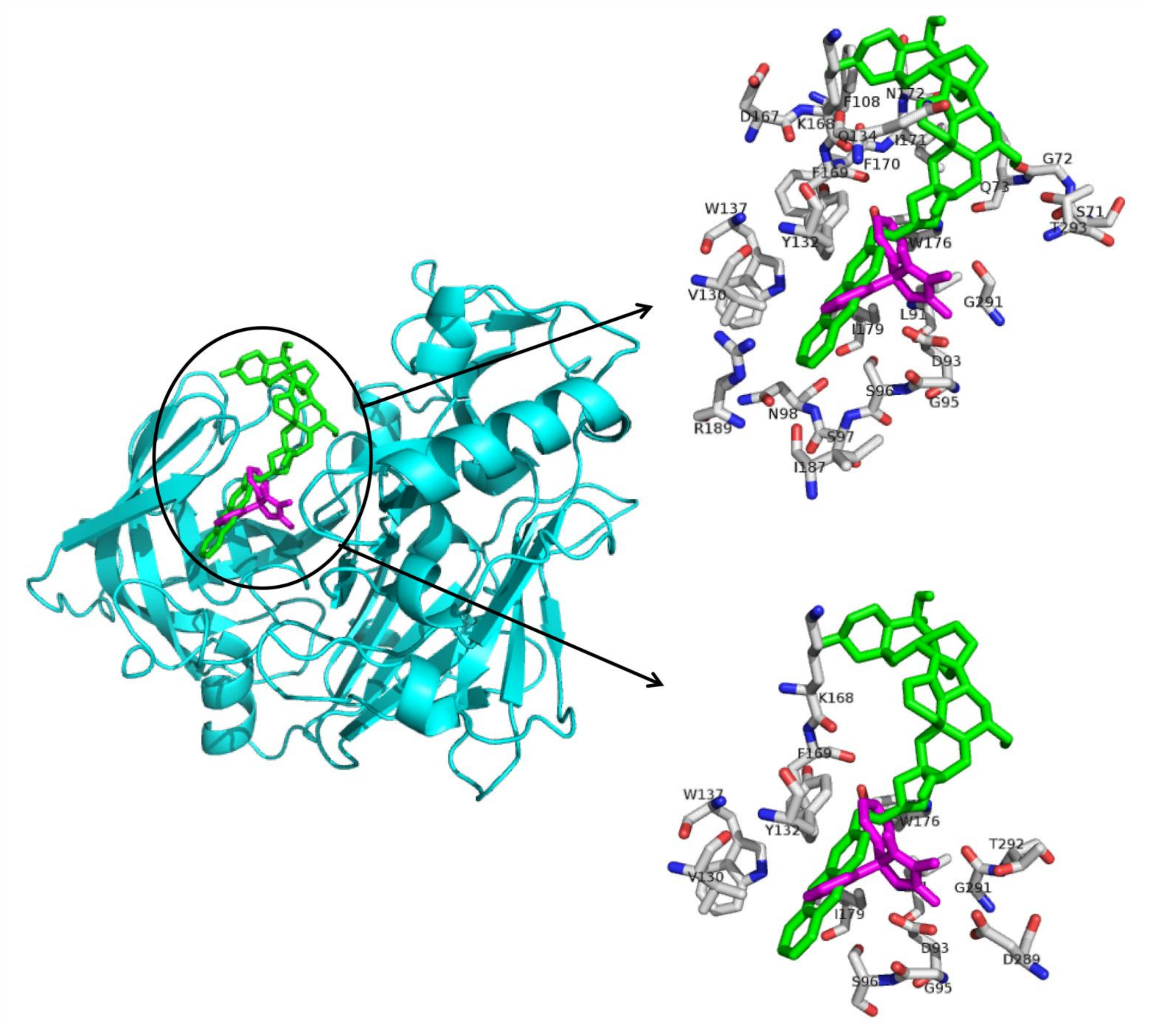

Supplementary Fig 18. The structures and binding model of ligands including best ligand and original ligand to the target protein BACE-1. The best ligand is green and the original ligand is magenta. The top panel shows the amino acid residues lying within 5 Å from the best ligand, and the bottom panel shows the amino acid residues lying within 5 Å from the original ligand.

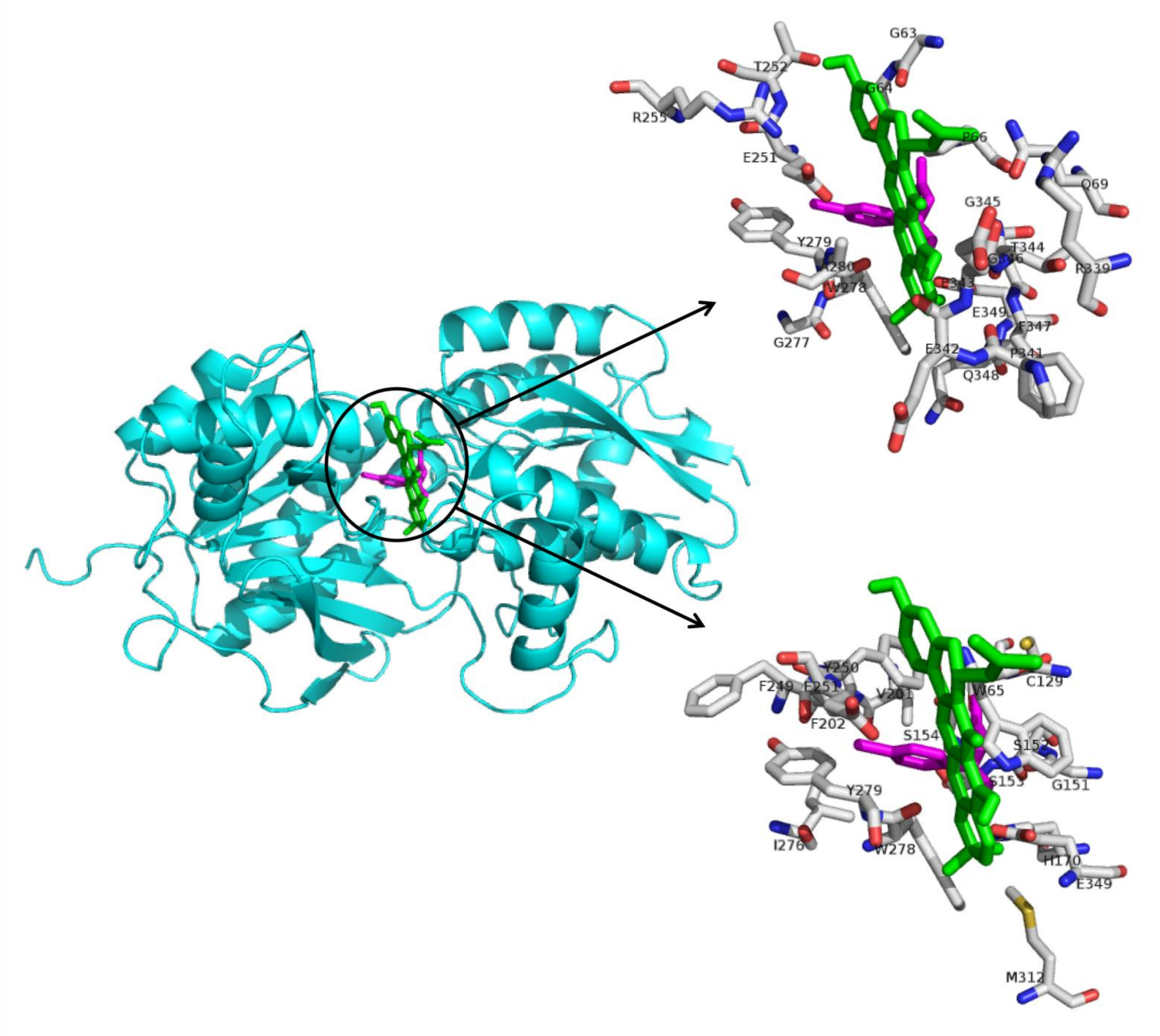

Supplementary Fig 19. The structures and binding model of ligands including best ligand and original ligand to the target protein GABA(B). The best ligand is green and the original ligand is magenta. The top panel shows the amino acid residues lying within 5 Å from the best ligand, and the bottom panel shows the amino acid residues lying within 5 Å from the original ligand.

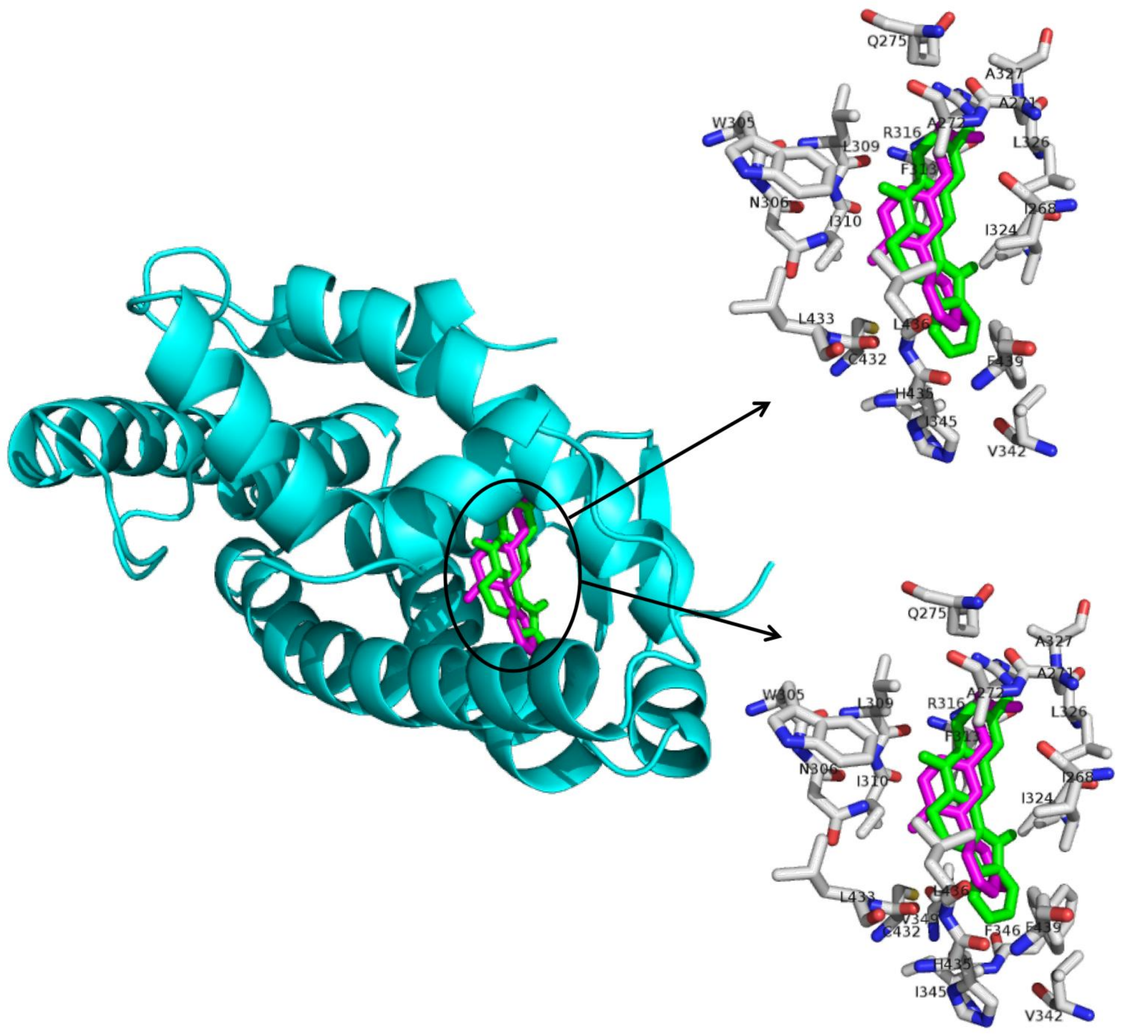

Supplementary Fig 20. The structures and binding model of ligands including best ligand and original ligand to the target protein RXR. The best ligand is green and the original ligand is magenta. The top panel shows the amino acid residues lying within 5 Å from the best ligand, and the bottom panel shows the amino acid residues lying within 5 Å from the original ligand.

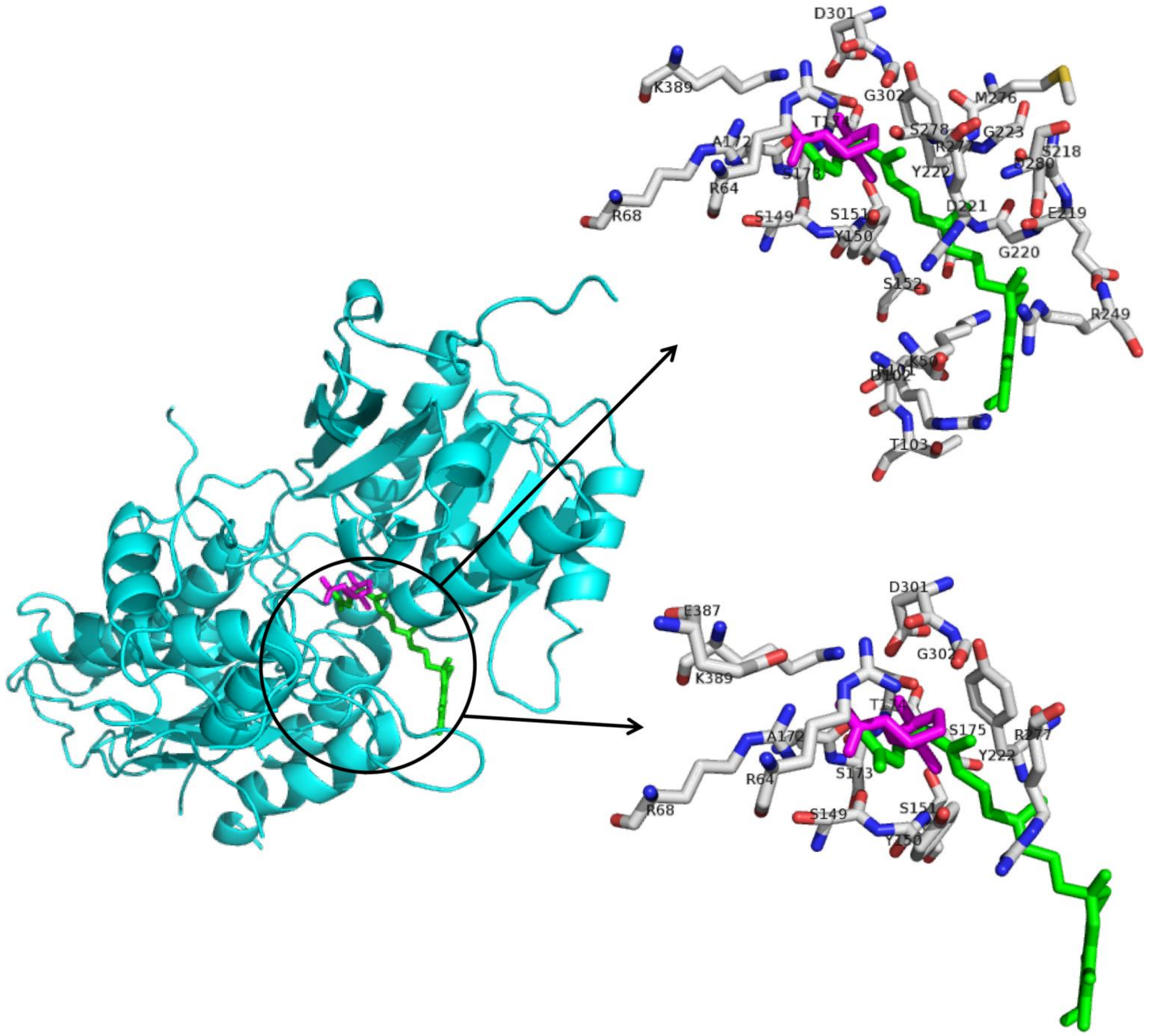

Supplementary Fig 21. The structures and binding model of ligands including best ligand and original ligand to the target protein MGLUR. The best ligand is green and the original ligand is magenta. The top panel shows the amino acid residues lying within 5 Å from the best ligand, and the bottom panel shows the amino acid residues lying within 5 Å from the original ligand.

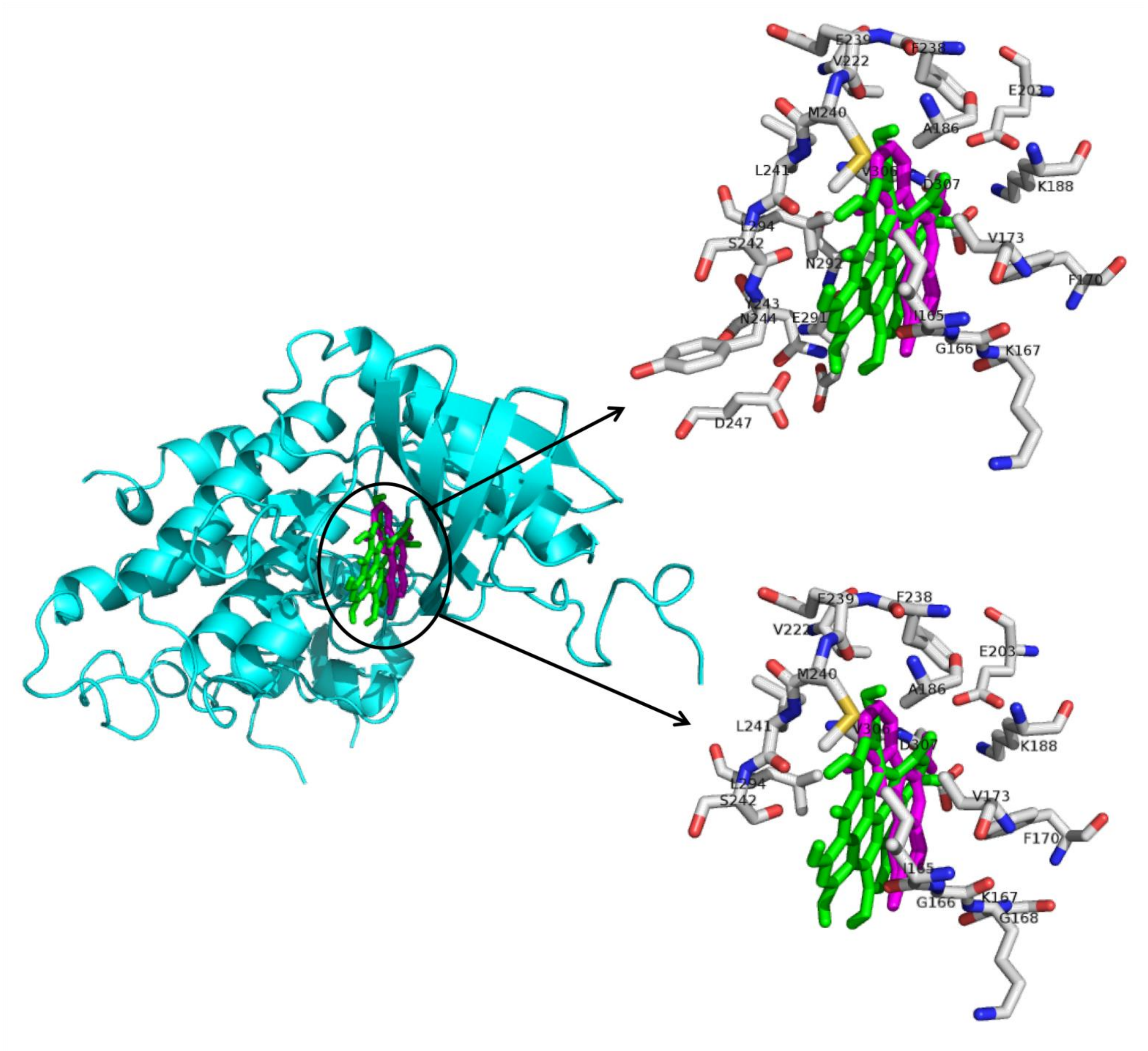

Supplementary Fig 22. The structures and binding model of ligands including best ligand and original ligand to the target protein DYRK1A. The best ligand is green and the original ligand is magenta. The top panel shows the amino acid residues lying within 5 Å from the best ligand, and the bottom panel shows the amino acid residues lying within 5 Å from the original ligand.

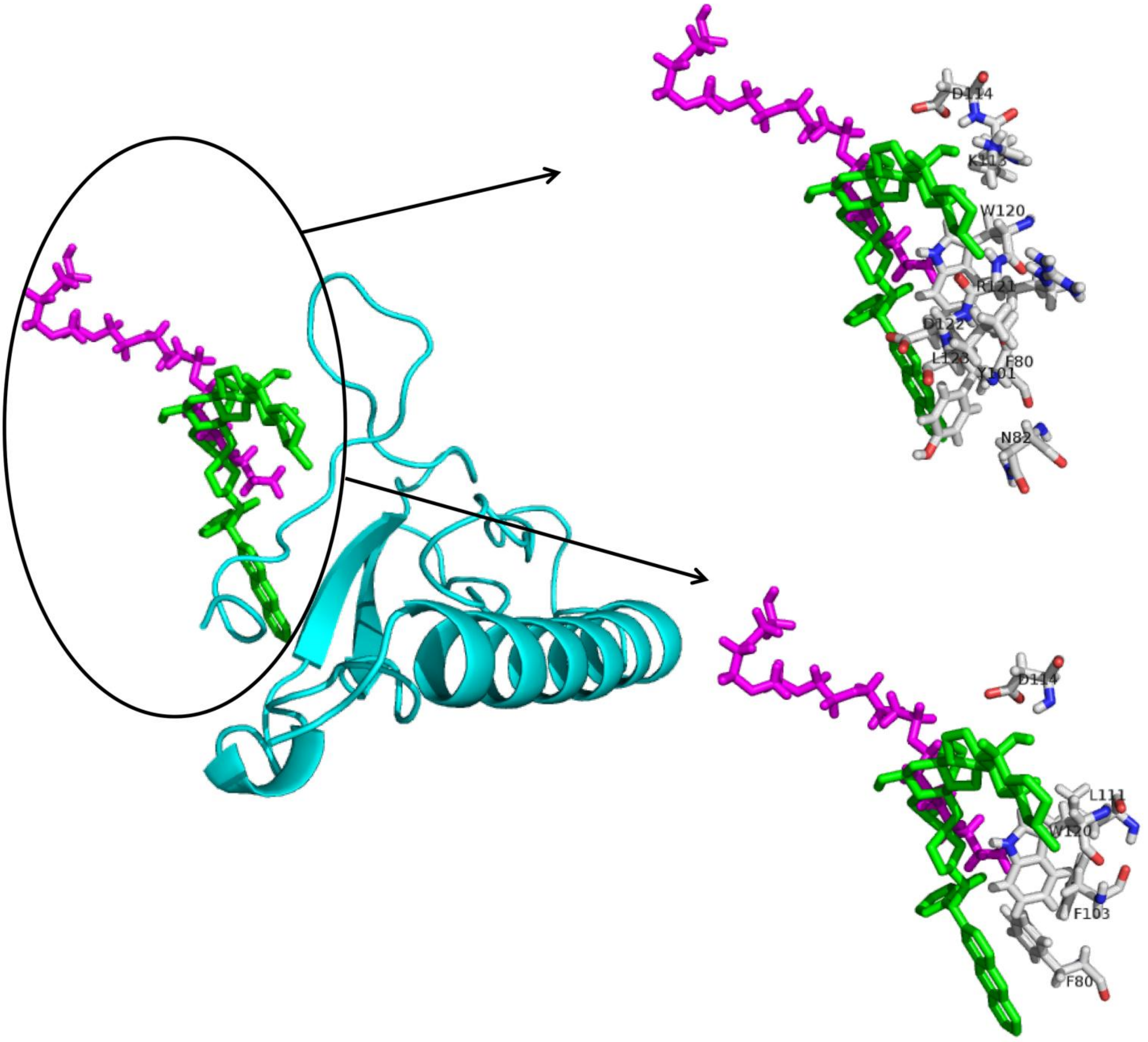

Supplementary Fig 23. The structures and binding model of ligands including best ligand and original ligand to the target protein GLP-1R. The best ligand is green and the original ligand is magenta. The top panel shows the amino acid residues lying within 5 Å from the best ligand, and the bottom panel shows the amino acid residues lying within 5 Å from the original ligand.

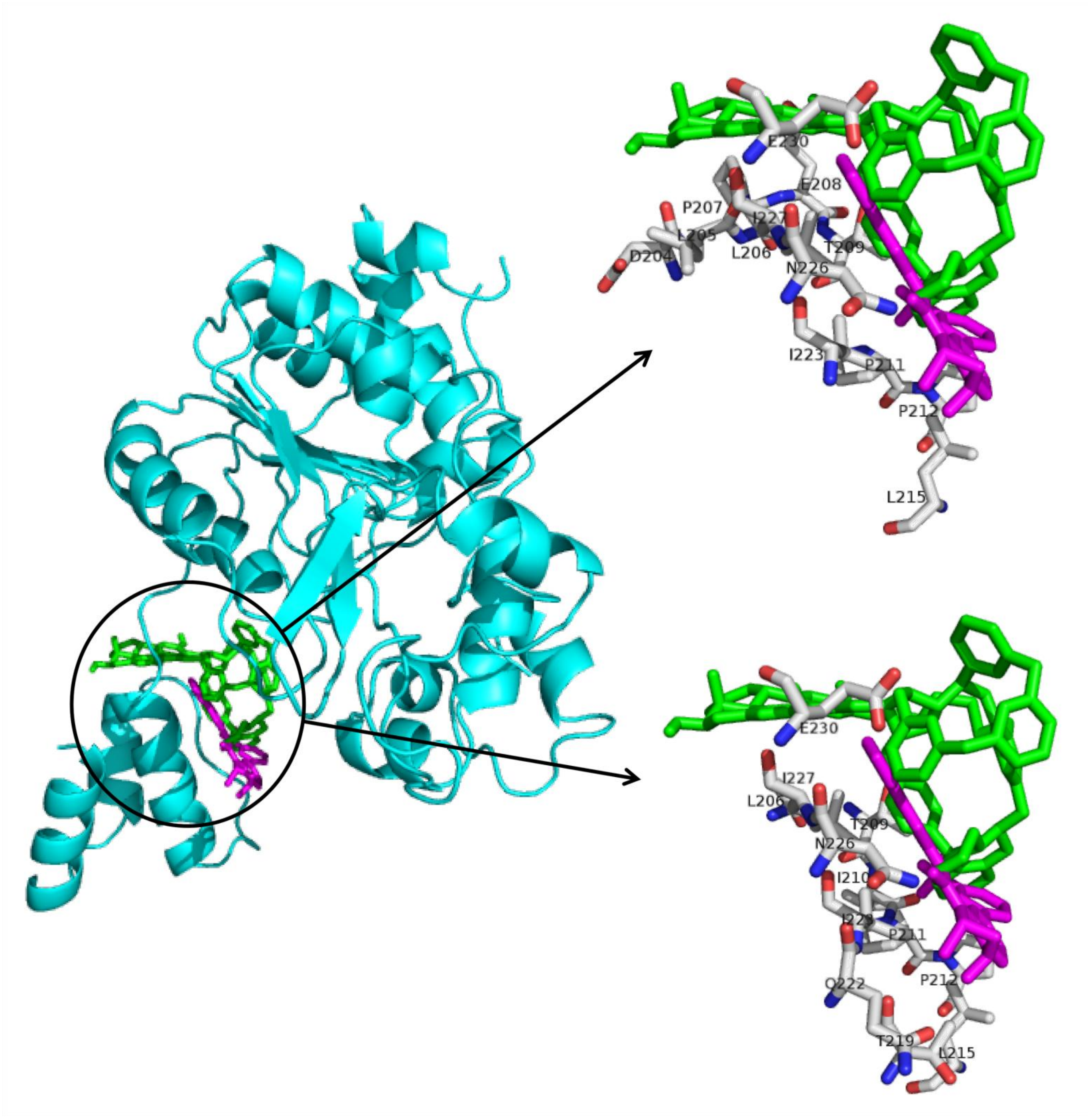

Supplementary Fig 24. The structures and binding model of ligands including best ligand and original ligand to the target protein SIRT1. The best ligand is green and the original ligand is magenta. The top panel shows the amino acid residues lying within 5 Å from the best ligand, and the bottom panel shows the amino acid residues lying within 5 Å from the original ligand.

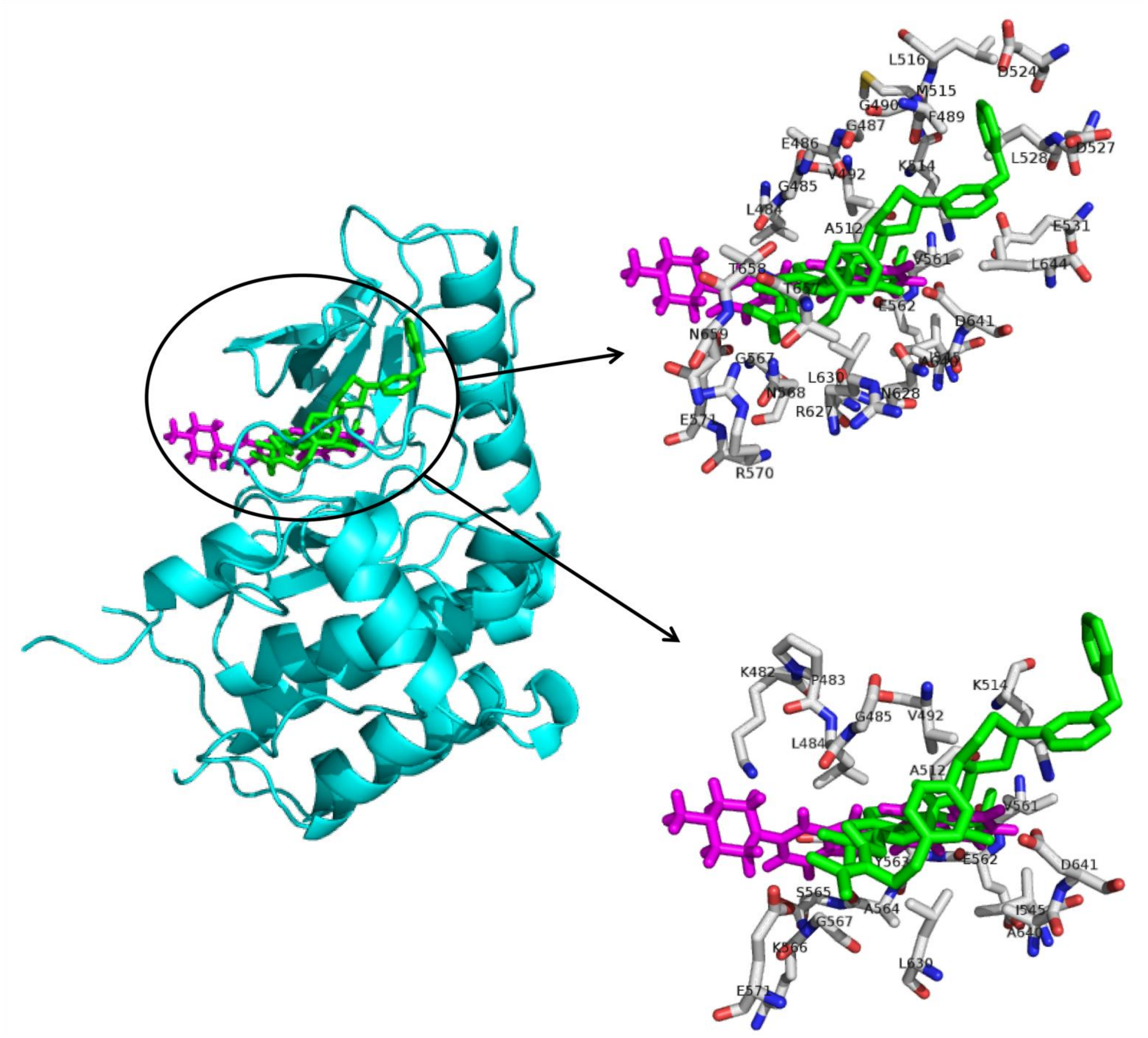

Supplementary Fig 25. The structures and binding model of ligands including best ligand and original ligand to the target protein FGFR1. The best ligand is green and the original ligand is magenta. The top panel shows the amino acid residues lying within 5 Å from the best ligand, and the bottom panel shows the amino acid residues lying within 5 Å from the original ligand.

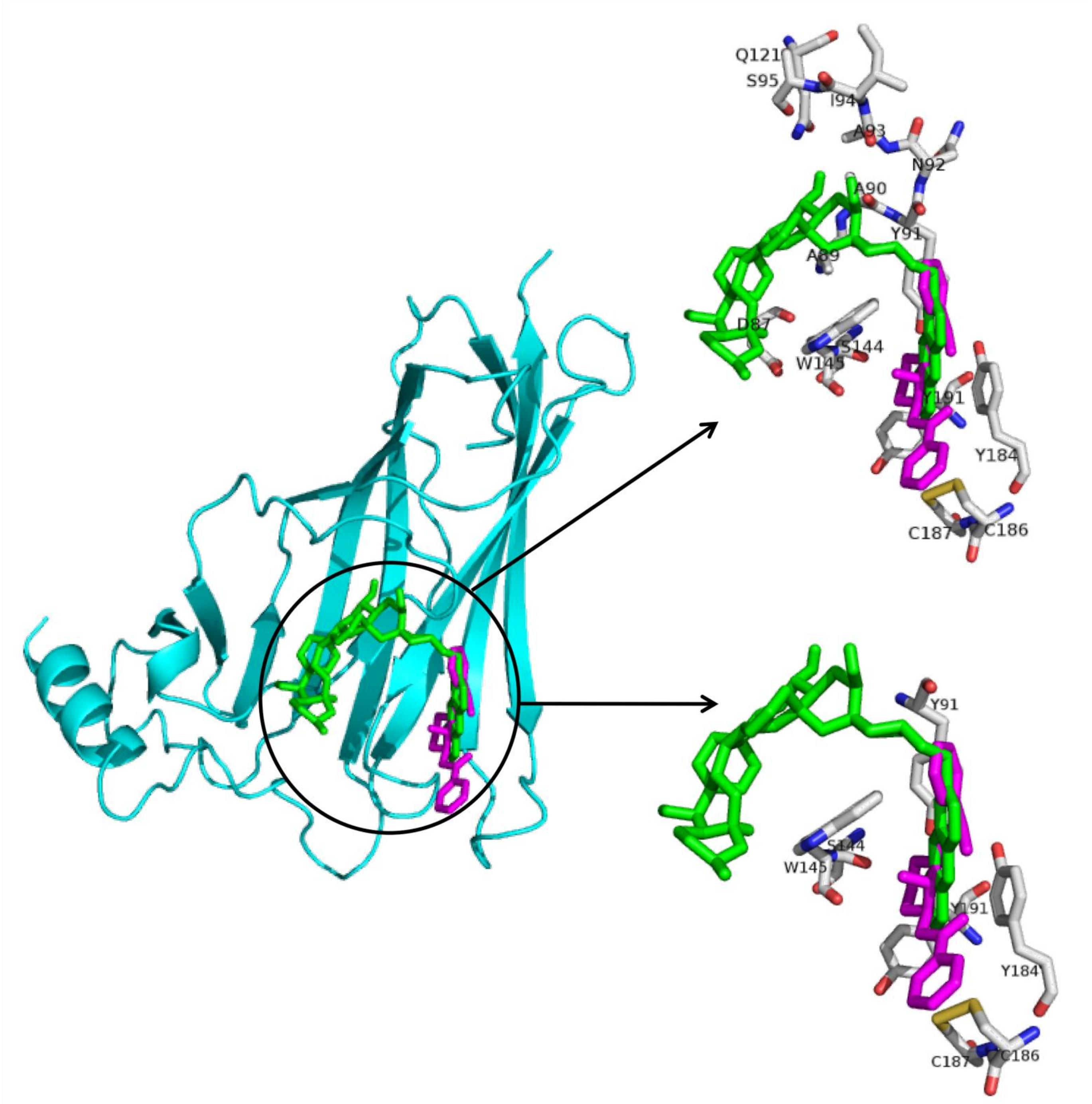

Supplementary Fig 26. The structures and binding model of ligands including best ligand and original ligand to the target protein α7NACHR. The best ligand is green and the original ligand is magenta. The top panel shows the amino acid residues lying within 5 Å from the best ligand, and the bottom panel shows the amino acid residues lying within 5 Å from the original ligand.

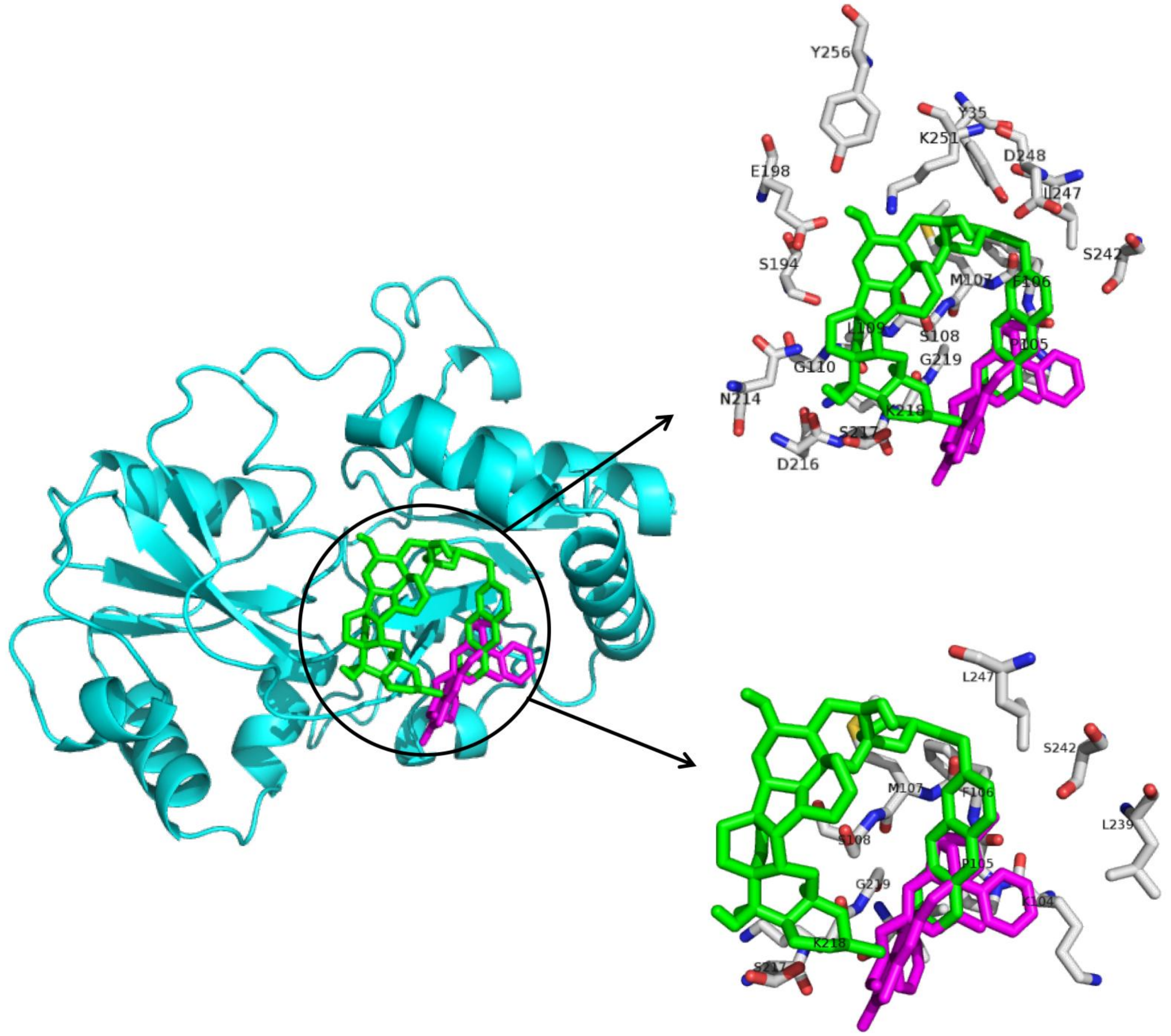

Supplementary Fig 27. The structures and binding model of ligands including best ligand and original ligand to the target protein AMPA. The best ligand is green and the original ligand is magenta. The top panel shows the amino acid residues lying within 5 Å from the best ligand, and the bottom panel shows the amino acid residues lying within 5 Å from the original ligand.

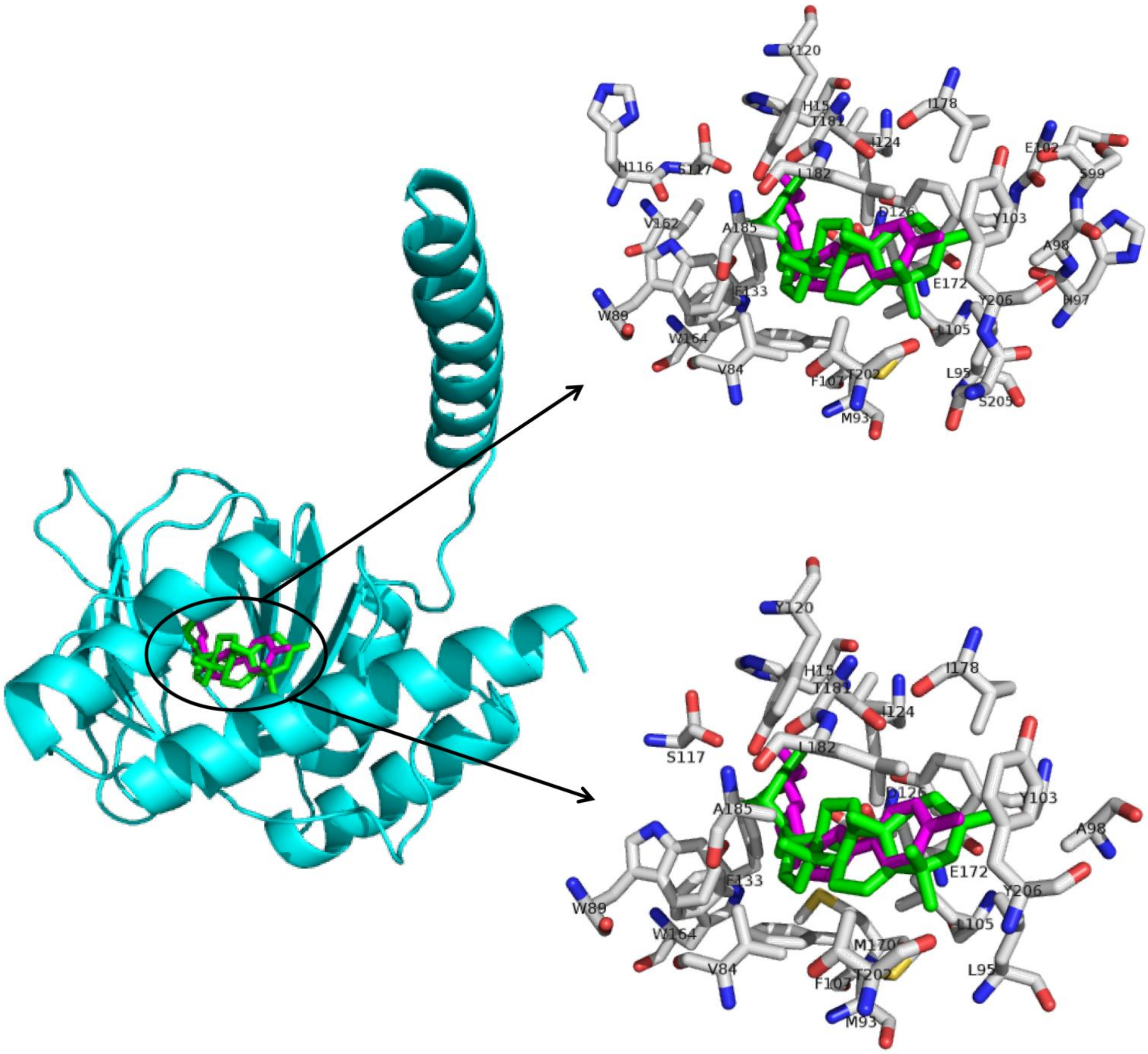

Supplementary Fig 28. The structures and binding model of ligands including best ligand and original ligand to the target protein SIG-1R. The best ligand is green and the original ligand is magenta. The top panel shows the amino acid residues lying within 5 Å from the best ligand, and the bottom panel shows the amino acid residues lying within 5 Å from the original ligand.

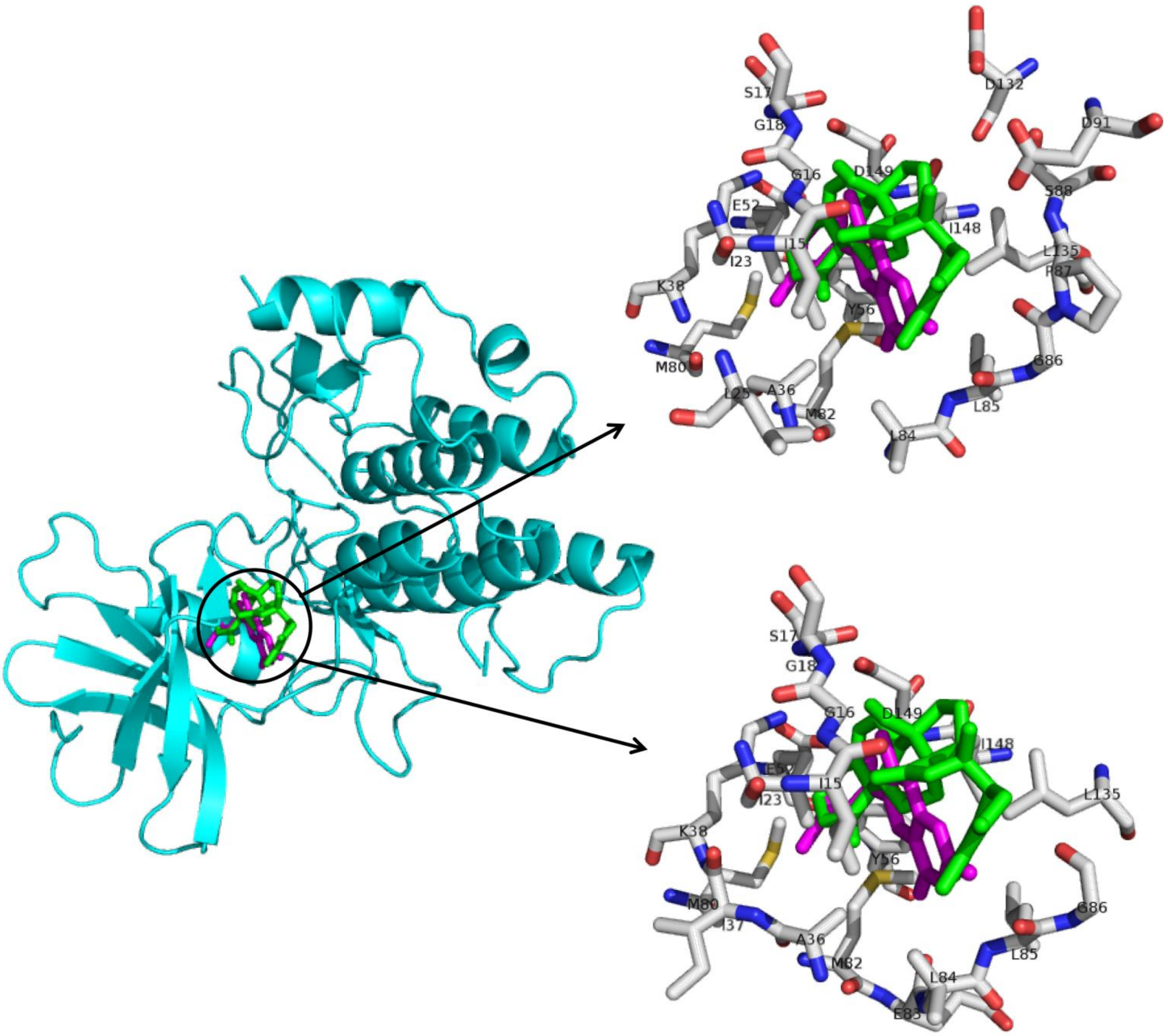

Supplementary Fig 29. The structures and binding model of ligands including best ligand and original ligand to the target protein CKI-δ. The best ligand is green and the original ligand is magenta. The top panel shows the amino acid residues lying within 5 Å from the best ligand, and the bottom panel shows the amino acid residues lying within 5 Å from the original ligand.

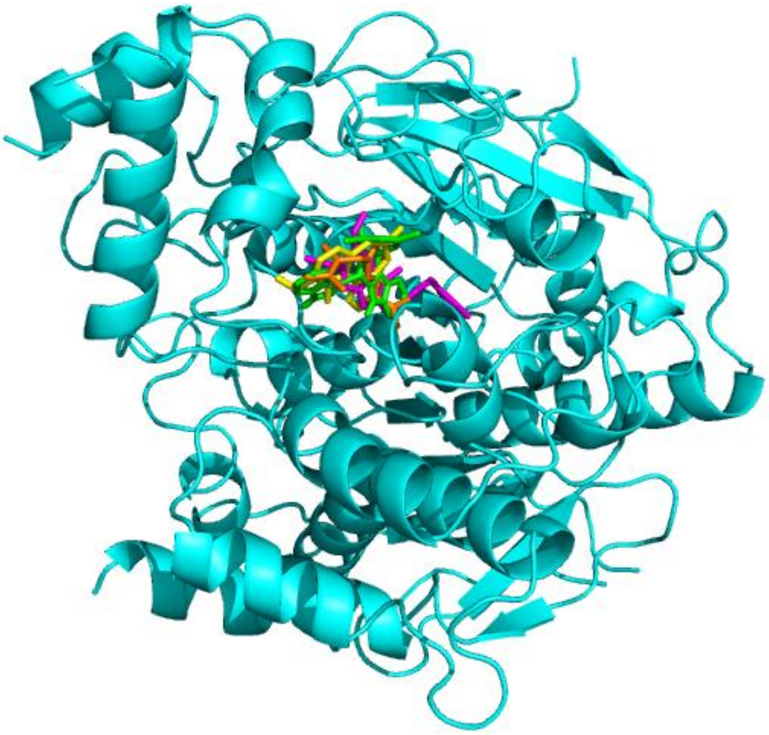

Supplementary Fig 30. The docking pose interaction between the target AchE and four ligands including the best binding TCM compound of AchE, Donepezil, Galantamine and Rivastigmine. The target AchE is cyan. The best binding TCM compound is green. Donepzil is magenta, Galantamine is yellow, and Rivastigmine is orange.

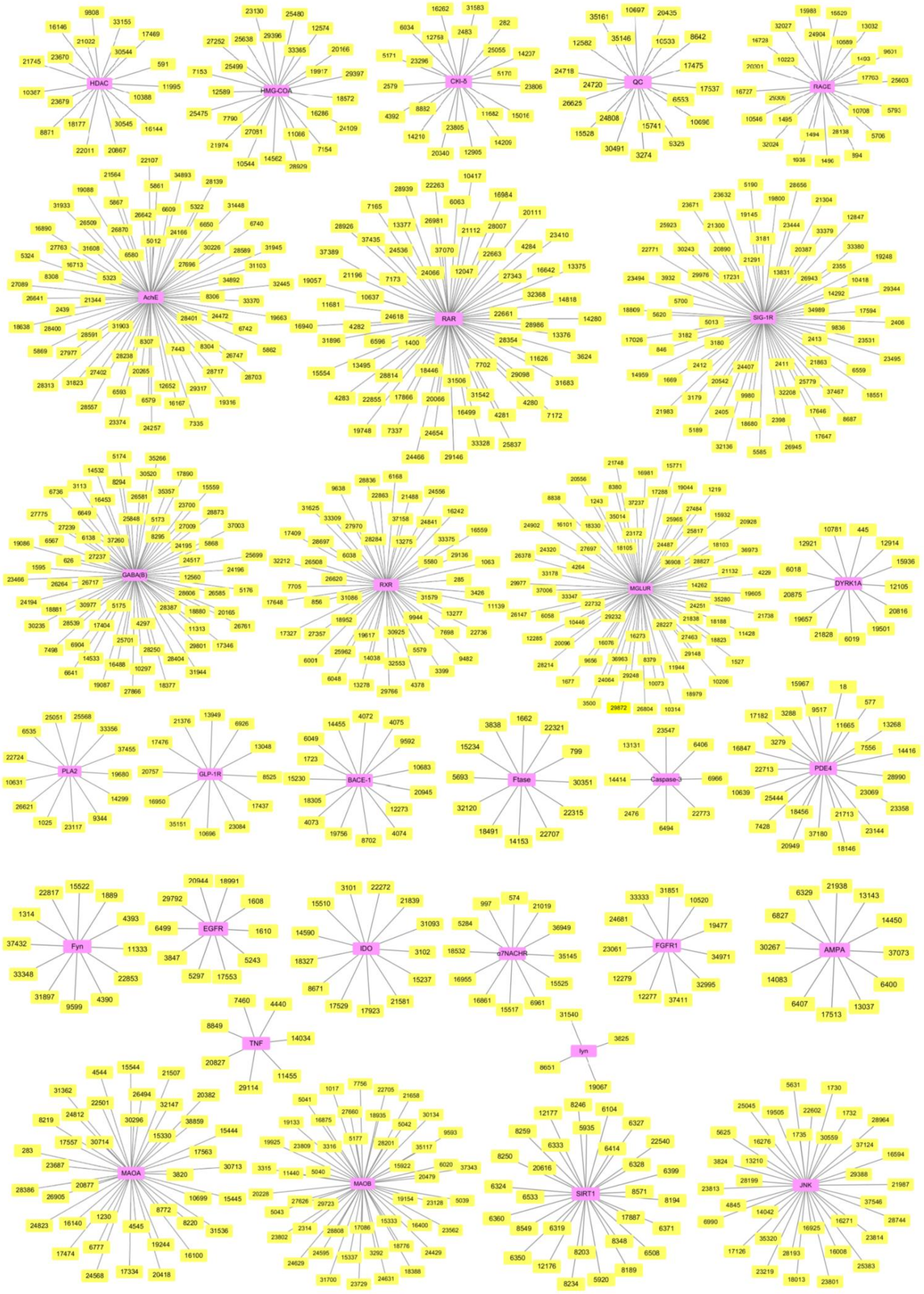

Supplementary Fig 31. The candidate anti-AD compounds with single target. The radial circle diagram shows single target compounds colored by yellow and their only target colored by pink.

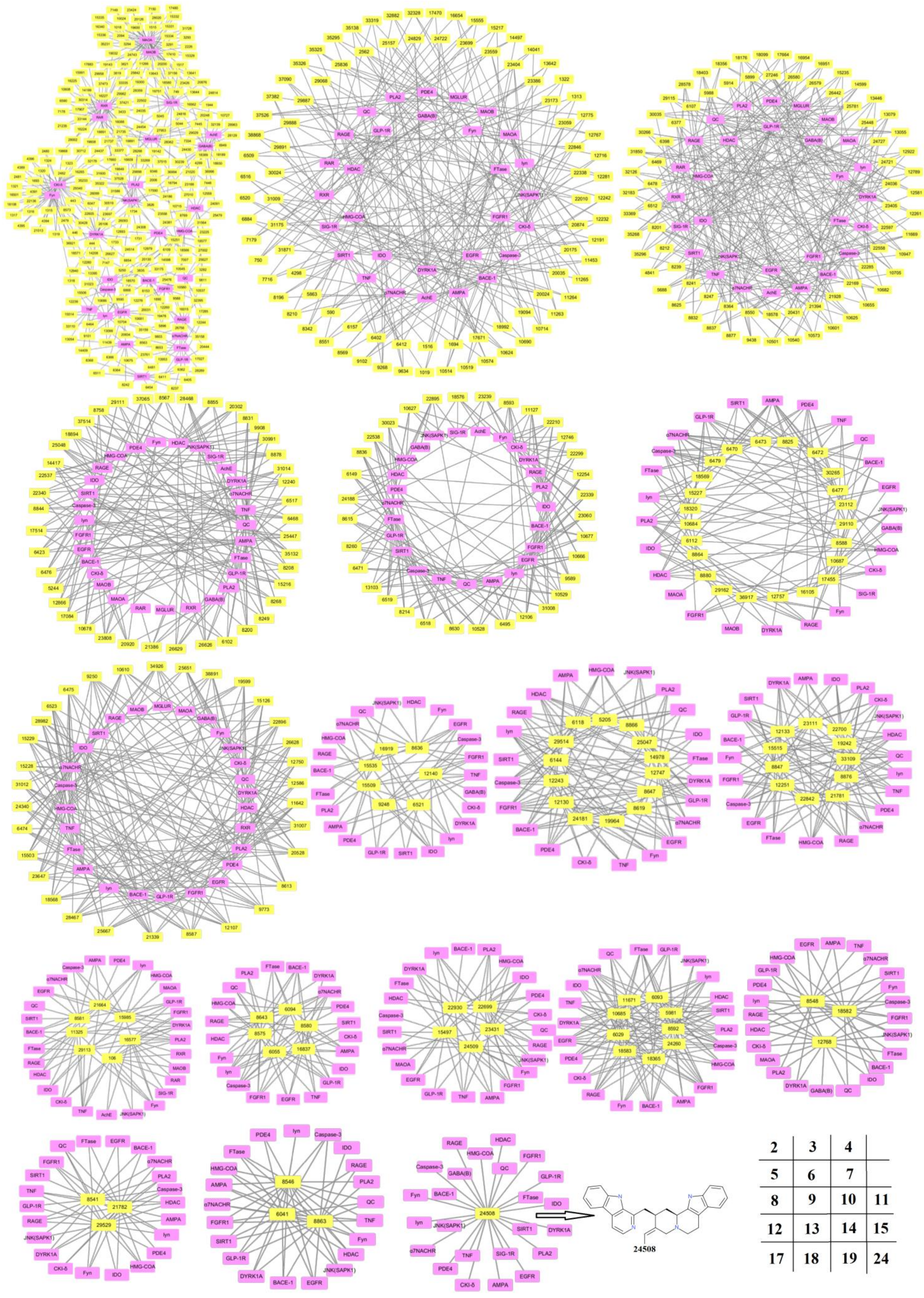

2	3	4	
5	6	7	
8	9	10	11
12	13	14	15
17	18	19	24

Supplementary Fig 32. Twenty networks of candidate anti-AD compounds with multiple targets. The compounds in each network have the same number of targets. In the bottom of right corner, the number means the targets number of compounds in each network. Compounds are displayed by small yellow boxes and targets displayed by pink boxes. The structure of compound 24508 is also shown.

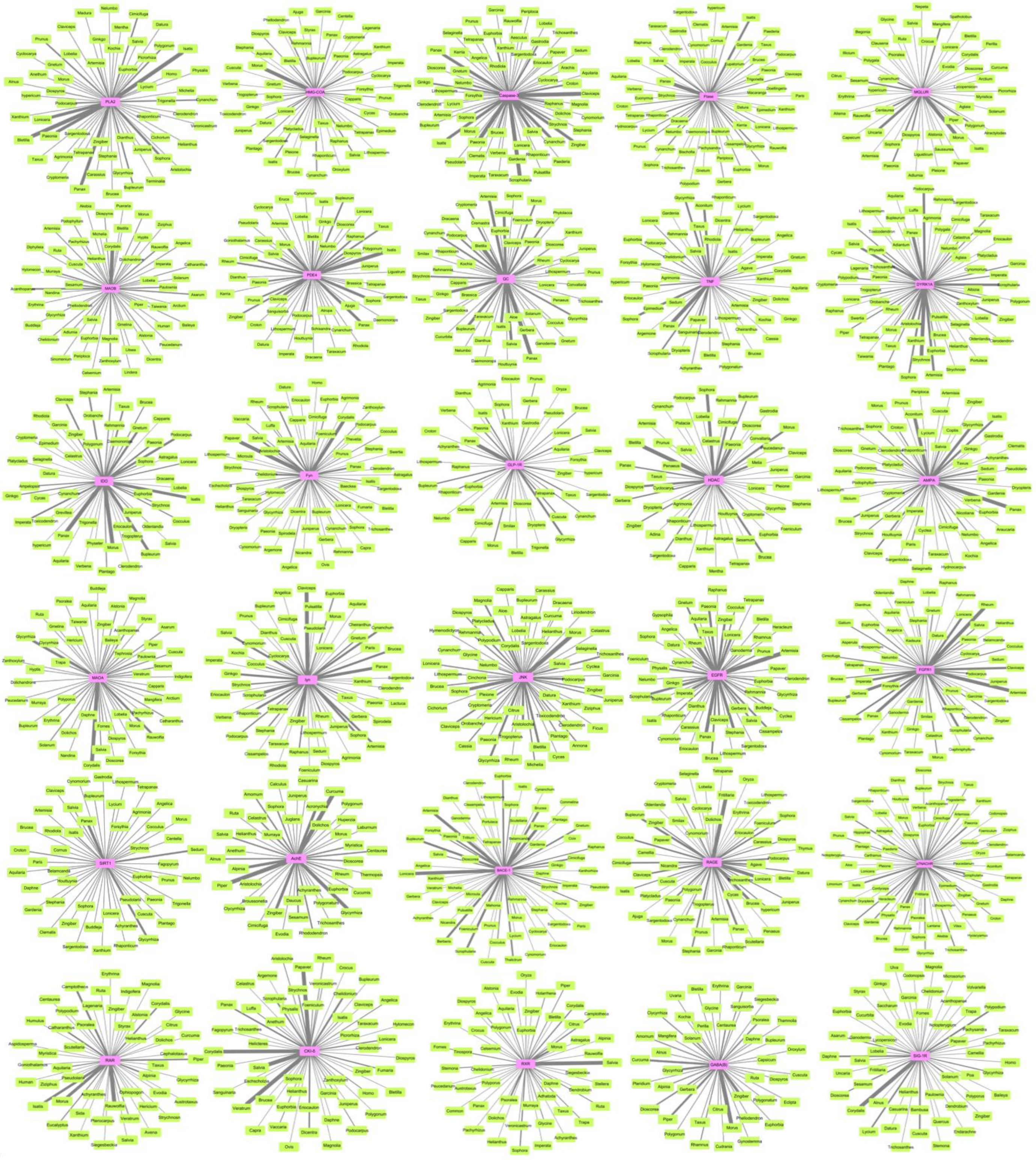

Supplementary Fig 33. Network of anti-AD targets and plants. Anti-AD targets actually connect with many different plants. Small boxes of green represent each plant marked their name. The line's thickness indicates the number of compounds, and the number ranges from 1 to 18.

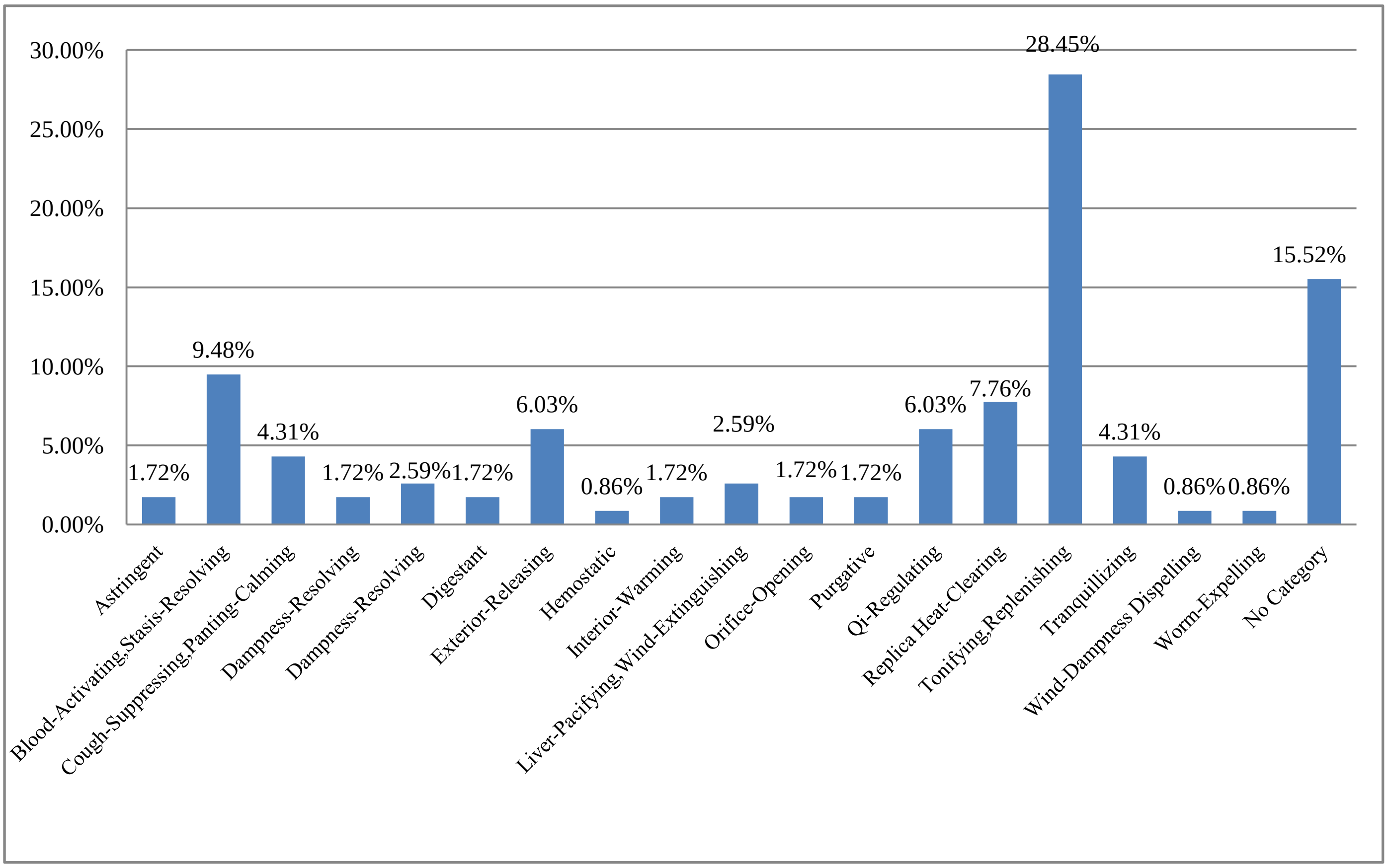

Supplementary Fig 34. Proportion of anti-AD plants with the different functional property. 141 anti-AD plants are classified in 19 types, one of which is not defined. Percentage indicates the proportion of the category.
